# Tissue-Based Proteomic Profiling in Patients with Hyperplasia and Endometrial Cancer

**DOI:** 10.3390/cells11132119

**Published:** 2022-07-05

**Authors:** Khalid Akkour, Ibrahim O. Alanazi, Assim A. Alfadda, Hani Alhalal, Afshan Masood, Mohthash Musambil, Anas M. Abdel Rahman, Moudi A. Alwehaibi, Maria Arafah, Ali Bassi, Hicham Benabdelkamel

**Affiliations:** 1 Obstetrics and Gynecology Department, College of Medicine, King Saud University, Riyadh 11461, Saudi Arabia; kakkour@ksu.edu.sa (K.A.); alhalalhani@gmail.com (H.A.); abassi@ksu.edu.sa (A.B.); 2 The National Center for Biotechnology (NCB), Life Science and Environment Research Institute, King Abdulaziz City for Science and Technology (KACST), Riyadh 11442, Saudi Arabia; ialenazi@kacst.edu.sa; 3 Proteomics Resource Unit, Obesity Research Center, College of Medicine, King Saud University, Riyadh 11461, Saudi Arabia; aalfadda@ksu.edu.sa (A.A.A.); afsmasood@ksu.edu.sa (A.M.); mthammitone@ksu.edu.sa (M.M.); modhialwehaibi@gmail.com (M.A.A.); 4 Department of Medicine, College of Medicine and King Saud Medical City, King Saud University, Riyadh 11461, Saudi Arabia; 5 Metabolomics Section, Department of Clinical Genomics, Center for Genome Medicine, King Faisal Specialist Hospital and Research Centre (KFSHRC), Riyadh 11211, Saudi Arabia; aabdelrahman46@kfshrc.edu.sa; 6 Department of Botany and Microbiology, College of Science, King Saud University, Riyadh 11461, Saudi Arabia; 7 Department of Pathology, College of Medicine, King Saud University, King Saud University Medical City, Riyadh 11461, Saudi Arabia; marafah83@gmail.com

**Keywords:** uterus, endometrial cancer, tissue, proteomics, hyperplasia, 2D-DIGE

## Abstract

Uterine cancers are among the most prevalent gynecological malignancies, and endometrial cancer (EC) is the most common in this group. This study used tissue-based proteomic profiling analysis in patients with endometrial cancer and hyperplasia, and control patients. Conventional 2D gel electrophoresis, followed by a mass spectrometry approach with bioinformatics, including a network pathway analysis pipeline, was used to identify differentially expressed proteins and associated metabolic pathways between the study groups. Thirty-six patients (twelve with endometrial cancer, twelve with hyperplasia, and twelve controls) were enrolled in this study. The mean age of the participants was 46–75 years. Eighty-seven proteins were significantly differentially expressed between the study groups, of which fifty-three were significantly differentially regulated (twenty-eight upregulated and twenty-five downregulated) in the tissue samples of EC patients compared to the control (Ctrl). Furthermore, 26 proteins were significantly dysregulated (8 upregulated and 18 downregulated) in tissue samples of hyperplasia (HY) patients compared to Ctrl. Thirty-two proteins (nineteen upregulated and thirteen downregulated) including desmin, peptidyl prolyl cis-trans isomerase A, and zinc finger protein 844 were downregulated in the EC group compared to the HY group. Additionally, fructose bisphosphate aldolase A, alpha enolase, and keratin type 1 cytoskeletal 10 were upregulated in the EC group compared to those in the HY group. The proteins identified in this study were known to regulate cellular processes (36%), followed by biological regulation (16%). Ingenuity pathway analysis found that proteins that are differentially expressed between EC and HY are linked to AKT, ACTA2, and other signaling pathways. The panels of protein markers identified in this study could be used as potential biomarkers for distinguishing between EC and HY and early diagnosis and progression of EC from hyperplasia and normal patients.

## 1. Introduction

Endometrial cancer (EC) is the sixth most common cancer in women globally [1], with the highest prevalence in North America and North Europe and the lowest in Southeast Asia and Africa [2]. EC includes 90% of all uterine cancers in women (primarily postmenopausal women) with a mean age of 60. The Bokhman’s dualistic model classifies EC into two main histological categories [3]. Estrogen-dependent type 1 (80–90% of endometrial cancer belongs to this category) is associated with endometrial hyperplasia, obesity, and metabolic abnormalities. Type II is estrogen-independent (accounts for 10–20% of EC cancers), high-grade, and clinically aggressive [4]. The currently available diagnostic procedures for endometrial cancer include a transvaginal ultrasound scan, outpatient hysteroscopy, and endometrial biopsy with histopathological analysis. However, histopathology fails to describe the molecular characterization of the tumor, which is why <20% of ECs initially assumed to be non-aggressive develop into aggressive and metastatic cancer in later stages of the patient’s life [5]. Thus, the low specificity and invasiveness of biopsy-like tools warrant the need to develop biomarkers that reflect the molecular profile of endometrial tumors in the early stages of the disease.

Proteomics, coupled with bioinformatics analysis, has emerged as a powerful tool for identifying biomarkers. As protein expression reflects physiological conditions better than the genes themselves, an untargeted approach using proteomic analysis will identify molecular fingerprints for the early detection of EC [6]. Biomarkers have recently been investigated using biological specimens such as blood, uterine aspirates, tissue biopsies, uterine lavage samples, and urine [7]. A few of the potential biomarkers reported from proteomics studies associated with EC include ANXA2, ABRACL, PGAM2, α-1-β glycoprotein, serum amyloid A, and heat-shock proteins (hsp10, hsp27, hsp70, hsp71) [7,8,9,10,11,12,13,14,15,16]. Although blood samples are easily obtained using minimally invasive techniques, they are limited by the low quantity of tumor-related signal circulation in the early stages of the disease [8]. Tissue samples are better for investigating biomarkers, because they are viable sources of cancer-derived proteins and are less complicated for proteomic analysis compared to blood, because of their lower protein dynamic range [9]. A considerable number of proteomic investigations on tissue and blood samples from EC [10,11,12,13,14,15,16] have been conducted. Most of them compared EC patients to healthy participants, leaving out crucial information on disease progression that could only be obtained by investigating patients with endometrial hyperplasia (progression stage/pre-cancer).

Only three studies have examined this aspect by considering the pre-cancer state (hyperplasia) in their research objective [10,17,18]. Byrjalsen et al. [18] performed a proteomic analysis of patients with hyperplasia and carcinoma, and reported the differential expression of cellular transport and chaperoning proteins: heat shock protein 27 kDa protein, heat shock 70 kDa protein, heat shock cognate 71 kDa protein, and serotransferrin. In 2011, Wang et al. [10] performed a quantitative proteomic analysis of serum samples from healthy women and patients with endometrial hyperplasia, complex endometrial hyperplasia, atypical endometrial hyperplasia, and endometrial carcinoma patients. Seven proteins (haptoglobin, SERPINC 1, alpha-1-antichymotrypsin, apolipoprotein A-IV, inter-alpha-trypsin inhibitor heavy chain H4, and histidine-rich glycoprotein) were differentially expressed in atypical endometrial hyperplasia. Recently, Ceylan et al. [17] compared the protein expression of complex atypical endometrial hyperplasia, endometrial carcinoma, and healthy endometrial tissues (D & C material) using two-dimensional difference gel electrophoresis (2D-DIGE). K2C8, UAP56, ENOA, ACTB, GRP78, GSTP1, PSME1, CALR, PPIA, PDIA3, and IDHc proteins were significantly expressed when comparing healthy participants and patients with cancer and complex atypical hyperplasia.

Although this study used the 2D-DIGE technique, pooled samples from different groups were used, which limited the statistical power of the study. Although these studies [10,17,18] employed proteomics as a tool, they could not clearly identify the differences between EC and hyperplasia. To the best of our knowledge, this study is the first to employ an untargeted proteomic approach to study the proteomic profiles of EC, hyperplasia (HY), and control (Ctrl) groups using tissue samples from Saudi women. We used 2D-DIGE, coupled with matrix-assisted laser desorption/ionization–time of flight (MALDI-TOF) mass spectrometry and bioinformatics analysis, to identify proteins that could act as potential biomarkers for the transition from healthy endometrial tissue to cancerous state via hyperplasia.

## 2. Materials and Methods

### 2.1. Ethical Approval and Consent to Participate

The study procedures and protocols were reviewed and approved by the institutional review board of the College of Medicine, King Saud University. Written informed consent was obtained from all the participants (IRB number: E-193622). This study was conducted at the Proteomics Resource Unit, Obesity Research Center, College of Medicine, and King Khalid University Hospital (KKUH), King Saud University, Riyadh, Saudi Arabia.

### 2.2. Study Design and Patient Selection

Patients attending the outpatient clinics of the Obstetrics and Gynecology–Oncology Department, King Khalid University Hospital, College of Medicine, King Saud University, in the age group of 46–75 “years” (age-matched) were recruited for the study. A total of 36 women were included. The primary assessment was performed during clinical appointments. Patients willing to participate in the study were recruited and informed consent was obtained. The patients were divided into EC, HY, and Ctrl groups based on the following inclusion criteria:

EC cases (*n* = 12): women diagnosed with EC with atypia tended toward metastatic changes, undergoing total hysterectomy. HY cases (*n* = 12): women diagnosed with hyperplasia that underwent total hysterectomy. Ctrl samples (*n* = 12): women with abnormal uterine bleeding, undergoing total hysterectomy for adenomyosis, fibroids, and other hormonal imbalances. The sample size was determined by conducting a power analysis using the Progenesis SameSpots non-linear dynamics statistical software to determine the minimum number of biological replicates required. Approximately 100 mg of tissue was excised from the endometrium of all 36 patients, including patients with HY and EC, and Ctrl, who underwent total hysterectomy. Frozen tissue sections were sent to the pathology department for histopathological examination (Supplementary Table S1). Fresh tissue samples were snap-frozen in liquid nitrogen and stored at −80 °C until analysis.

### 2.3. Tissue Protein Extraction

Proteins were extracted from endometrial tissue samples using a T25 digital ULTRA TURRAX homogenizer (IKA, Staufen, Germany) directly in lysis buffer (0.5 mL, pH 8.8, 30 mM Tris-HCl, 7 M urea, 2 M thiourea, 2% CHAPS; we used 4% CHAPS and a 1X protease inhibitor mix) on ice. The suspension was shaken for 1 h at room temperature and then sonicated (Microsonicator, Qsonica Sonicators, Newtown, CT, USA; 30% pulse, two intervals of 1 min each, separated by a 1 min gap). Fifty millimolar dithiothreitol (DTT) was added and the protein extracts were centrifuged (20,000× *g*, 40 min, 4 °C). Contaminants were removed, and supernatants were cleaned by precipitation using a 2D clean-up kit according to the manufacturer’s protocol (GE Healthcare, Danderyd, Sweden) [19,20].

### 2.4. CyDye Labelling, Two-Dimensional (2D) Electrophoresis, and Image Scanning

The protein pellets were each resuspended in labelling buffer (7 M urea, 2 M thiourea, 4% CHAPS, 30 mM Tris) and the pH was adjusted to 8.5. Protein concentrations were determined in triplicates using a 2D-Quantkit (GE Healthcare, Danderyd, Sweden). The proteins were labeled with CyDye™ DIGE Fluor minimal dye (400 pmol/50 μg) (GE Healthcare, Danderyd, Sweden) according to the manufacturer’s recommendations, as previously described by our group [19,20,21,22]. Briefly, for each sample, 50 μg of protein was incubated (30 min on ice in the dark) with 400 pmol of Cy3 or Cy5 freshly dissolved in anhydrous dimethyl formamide (DMF). The reaction was quenched by adding lysine (1.0 μL, 10 mM, and 10 min on ice in the dark). Each sample was covalently labelled with a fluorophore, either Cy3 or Cy5. A pooled internal standard consisting of 50 μg of total protein from each of the 36 samples was labelled with Cy2. The labelled samples were combined according to the experimental design (Supplementary Table S2) and run on the same gel for comparison, as previously described [19,20,21,22]. First-dimension analytical gel electrophoresis was performed as previously described [19,20,21,22]. Eighteen Immobiline Dry Strips (24 cm, pH 3–11; GE Healthcare, Danderyd, Sweden) were rehydrated passively (30 V, 12 h). This was followed by isoelectric focusing using an Ettan IPGphor IEF unit (GE Healthcare, Danderyd, Sweden). Focusing was performed at 20 °C and 50 μA per strip, according to the following steps and hold sequence: (1) 500 V for 1 h, (2) 1000 V for 1 h, (3) 8000 V for 3 h, and (4) 8000 V up to 45,000 V for 22 h. The IPG strips were then stored at −80 °C until separation in the second dimension was performed. Before the second-dimension separation, the IPG strips were equilibrated (15 min, RT, gentle stirring, 5 mM Tris–HCl, pH 8.8, 6 M urea, 30% glycerol, 2% SDS, 65 mM DTT). The strips were equilibrated for an additional 15 min in the same solution containing 250 mM iodoacetamide. Polyacrylamide gradient gels (5–20%) were prepared on low-fluorescence glass. Second-dimension sodium dodecyl sulfate polyacrylamide gel electrophoresis (SDS-PAGE) was performed (Ettan DALT six vertical units, GE Healthcare, Danderyd, Sweden; 15 °C, 1 W per gel for 1 h, and then 2 W per gel until the bromophenol blue dye front reached the bottom of the gel). After SDS-PAGE electrophoresis, the gels were scanned with Sapphire Biomolecular Imager (Azure Bio systems, Dublin, OH, USA) and digitalized via the image analysis software Sapphire Capture system (Azure Biosystems, Dublin, OH, USA). Preparative gels were prepared using total protein (1 mg) obtained from a pool of equal protein amounts from the 36 endometrial tissue samples (12 EC, 12 HY, and 12 Ctrl). Gels were stained for 5 days, and the stained gels were briefly rinsed with Milli-Q water and stored until the spots could be picked and identified using MS, as previously described [19,20,21,22].

### 2.5. Statistical Analysis

The Progenesis SameSpots software (Nonlinear Dynamics, Newcastle upon Tyne, UK) was used to analyze 2D-DIGE gel images using an automated spot detection method. The software included gel warping, DIGE normalization, and comparison modules. All gel images were aligned to the reference gel and overlaid to ensure that no data were lost. The software calculated the normalized volume (NV) of each spot on each gel from the Cy3 (or Cy5) to the Cy2 spot volume ratio. A log transformation of the spot volumes was performed to generate the normally distributed data. Log-normalized volume (LNV) was used to quantify differential expression. The EC, HY, and Ctrl groups were compared directly, and fold difference values and *p*-values were calculated using one-way analysis of variance. All spots were pre-filtered and manually checked before applying statistical criteria (ANOVA, *p* ≤ 0.05). Normalized spot volumes, instead of spot intensities, were used for statistical processing. Only spots that fulfilled the above statistical criteria were subjected to the MS analysis.

### 2.6. Protein Identification Using MALDI-TOF Mass Spectrometry

Coomassie-stained gel spots were excised manually, washed, and digested according to previously described methods [18,19,20,21]. A mixture of tryptic peptides (0.8 μL) derived from each protein was spotted onto a MALDI target (384 MTP Anchorchip; 800 μm Anchorchip; Bruker Daltonics, Bremen, Germany). MALDI-MS/MS spectra were obtained using an UltraflexTerm TOF mass spectrometer equipped with a LIFT-MS/MS device (Bruker Daltonics) at reflector and detector voltages of 21 kV and 17 kV, respectively, as was described before [18,19,20,21]. Peptide mass fingerprints (PMFs) were calibrated against a standard (peptide calibration standard II, Bruker Daltonics). The PMFs were assessed using the Flex Analysis software (version 2.4, Bruker Daltonics). MS data were interpreted using BioTools v3.2 (Bruker Daltonics). The peptide masses were searched using the Mascot search algorithm (v2.0.04, updated on 9 May 2021; Matrix Science Ltd., London, UK). Identified proteins were accepted as correct if they had a Mascot score greater than 56. The Mascot significance score was calculated using the formula protein score = −10 * Log(P), where P is the probability that the observed match is a random event; protein score greater than 56 was considered significant (*p* ≤ 0.05). ID proteins with low scores were excluded, as they were mostly random matches and insignificant (*p* > 0.05). Not all spots of interest could be identified because some proteins were of low abundance and did not yield sufficiently intense mass fingerprints; other spots were mixtures of multiple proteins [19,20,21,22].

### 2.7. Bioinformatics Analysis

Ingenuity Pathway Analysis (IPA version 9.0; Ingenuity Systems, Redwood City, CA, USA) was used to analyze protein interaction networks and the functions of the tissue proteins differentially expressed in EC, HY, and Ctrl samples. Ingenuity pathway analysis (IPA) software maps UniProt IDs into the ingenuity knowledge base, which is the largest manually curated resource combining information from all published scientific studies. This software assists in determining functions and pathways that are strongly correlated with the MS-generated protein list by overlaying the experimental expression data onto networks constructed from published interactions. Additionally, the identified proteins were classified into different categories according to their molecular function and biological process using the protein analysis through evolutionary relationships (PANTHER) classification system (http://www.pantherdb.org, accessed on 22 July 2021).

### 2.8. Validation of Results Using LC- MS/MS (MRM)

Six different proteins were selected from the proteomics profile, where at least one signature peptide per protein was identified using the criteria described previously, using Skyline Software v21 (MacCross Lab, Seattle, WA, USA) [23,24]. The suggested MRM transitions were exported to a Triple-Quadrupole-Tandem Mass spectrometer (XEVO TQmicro, Waters Corporation, Milford, MA, USA). A control-extracted sample was used to evaluate the calculated transitions and to optimize the collision energy and column retention time. The patient samples were digested with trypsin and solid-phase extracted using the standard protocol reported by Galal et al., 2021 [25]. The extracted tryptic peptides were separated using an Acquity Ultra-Performance Liquid Chromatography (UPLC) AQUITY BEH C18, 1.7 μm, 2.1 mm × 100 mm column (at 25 °C) at a mobile phase flow rate of 0.3 mL/min over a total run time of 12 min (solvent A: 0.1% formic acid in H_2_O; solvent B: 0.1% formic acid in acetonitrile). The gradient profile for solvent A (0.1% formic acid in H_2_O) was 90% for 1 min, followed by a linear gradient to 10% over 10 min, which was then held at 10% for 1 min before returning to 90% in 2 min. For positive-mode mass spectrometric resolution, the eluted peptides were subjected to electrospray ionization (ESI). The source desolvation temperature was set at 450 °C, desolvation gas flow was set at 700 L/Hr, cone gas flow at 50 L/Hr, MS capillary source voltage at 1.98 KV, and cone source at 47 V. The total run time for each sample was 12 min at a mobile phase flow rate of 0.3 mL/min following the gradient table. The samples were stored in an autosampler at 4 °C, with an injection volume of 5 μL. During the run, frequent intermediate washing steps were performed to minimize the sample carryover. The experimental conditions are summarized in Supplementary Table S4.

## 3. Results

### 3.1. Proteomic Analysis and Identification of Differentially Expressed Proteins Using 2D-DIGE Analysis

**Figure 1:**
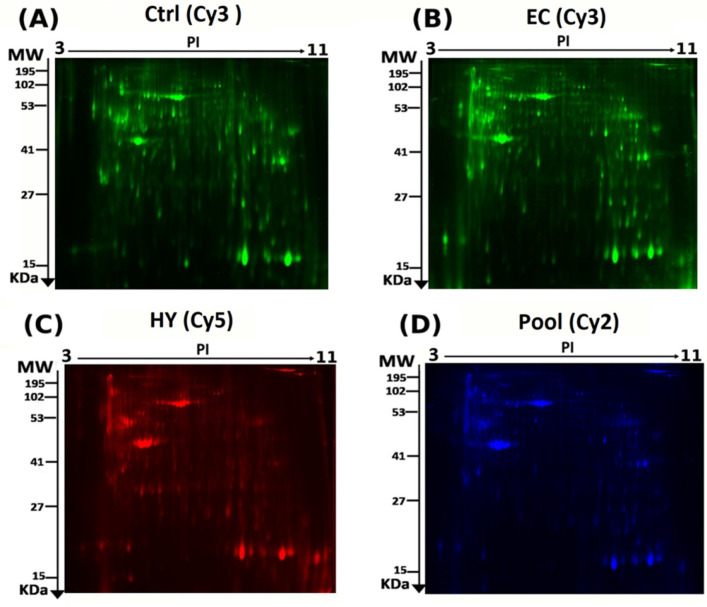
Representative fluorescent protein profiles obtained using 2D-DIGE containing: (**A**) Ctrl (sample no: Ctrl1) labeled with Cy3 (green), (**B**) EC (sample no: EC2) labeled with Cy3 (green), (**C**) HY (sample no: HY2) labeled with Cy5 (red), and (**D**) pooled internal control labeled with Cy2 (blue).

To identify the differential protein expression between the EC, HY, and Ctrl tissues (36 tissue samples analyzed in 18 gels), we performed 2D-DIGE and MALDI-TOF MS. Representative fluorescent protein profiles are shown in Figure 1; Ctrl (Figure 1A) and EC samples (Figure 1B) labeled with Cy3, HY samples labeled with Cy5 (Figure 1C), and pooled internal control labeled with Cy2 (Figure 1D). Merged 2D-DIGE gel images of Cy3/Cy5 between EC and Ctrl are shown in Supplementary Figure S1A, and merged 2D-DIGE gel images of Cy3/Cy5 between CA and HY are shown in Figure S1B. Automated image analysis detected 1260 spots on the gels, of which 148 were statistically significant (ANOVA, *p* ≤ 0.05) between the EC, HY, and Ctrl groups (Supplementary Figure S2). The spot patterns were reproducible across all 18 image gels, which were aligned and analyzed further. Normalization across the complete set of gels and quantitative differential analysis of protein levels were achieved using an internal standard with Cy2-labelling. The 148 spots showing statistical significance among the three groups were then manually excised from the preparative gel for protein identification and analyzed using MALDI-TOF MS. 

PMFs successfully identified 87 significant abundant proteins (*p*-value ≤ 0.05, cutoff ≥ 2.0 fold) excised from the preparative gel, and MALDI-TOF mass spectrometry identified 55 spots as unique proteins (Table 1, Supplementary Table S3). The sequence coverage of the proteins identified using PMF ranged between 11 and 89%. In a few cases, variants of the same protein were found at several locations on the gel (Table 1, Figure S2). Proteins ACTA, DESM, and TAGL3 (complete list provided in Table 1) were found in more than one spot on the gels, which could be associated with post-translational-modification-associated cleavage by enzymes or the presence of different protein species.

### 3.2. Principal Component Analysis

**Figure 2:**
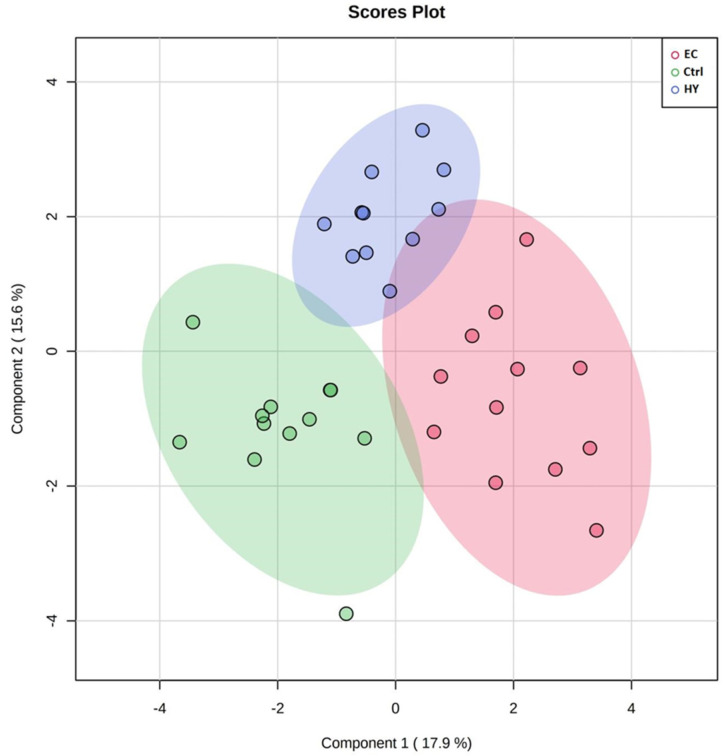
Two−dimensional principal component analysis (2D PCA) scores plots of significant differentially expressed proteins identified between the three groups of the study. The Ctrl, HY, and EC samples are represented as green, blue, and red circles, respectively.

PCA was used to visualize each study group and detect outliers. The score plots obtained for all the three study groups are shown in Figure 2. The samples were colored according to their group. The PCA model demonstrating that the Ctrl, HY, and EC groups clustered in a two-dimensional score plot indicated that the proteomics profile was significantly different between these three groups. In Figure 2, the primary source of variance (PC1, explaining 17.9% of the variance) allows separation of the EC group (red circles) and HY (blue circles) from the Ctrl samples (green circles). Simultaneously, component 2 (15.6%) describes the variation between two groups of HY (blue circles) and Ctrl.

### 3.3. The Overall Proteomic Analysis

**Figure 3:**
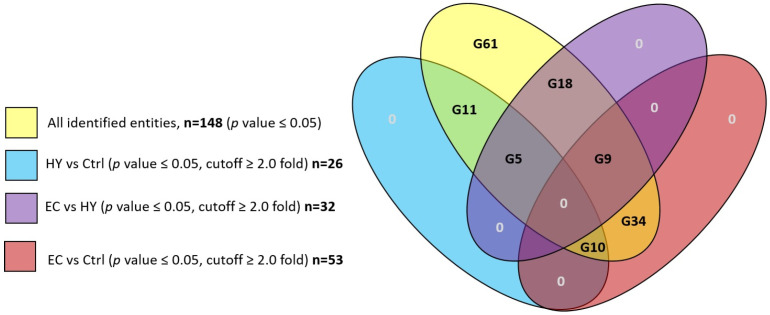
Venn diagram showing overlap among the groups HY vs. Ctrl, EC vs. HY, EC vs. Ctrl with overall detected proteins.

A total of 148 protein spots were differentially expressed with statistical significance (*p* ≤ 0.05, gel analysis) between the three study groups based on analysis carried out using the Progenesis SameSpots non-linear dynamics statistical software. Detailed protein expression data were analyzed using Multiple Professional Profiler (MPP) software (Agilent Inc., Santa Clara, CA, USA). The analysis revealed 61 (G61) of them to be statistically non-significant (cut off < 2.0) and 87 protein spots to be significant in multiple binary analyses (*p*-value ≤ 0.05, cutoff ≥ 2.0 fold). The other groups analyzed included HY vs. Ctrl group with 26 significant proteins (G26 included subgroups: G11, G5, and G10), EC vs. HY group with 32 significant proteins (G32 included subgroups: G18, G9, and G5), and EC vs. Ctrl with 53 significant proteins (G53 included subgroups: G34, G10, and G9) as shown in Figure 3.

### 3.4. Cancer vs. Control Proteomic Pattern

EC vs. Ctrl comparison shows 53 proteins with statistically significant changes in abundance (*p*-value ≤ 0.05, cutoff ≥ 2.0 fold) as shown in Table 1, Figure 3. Among this 53, included subgroups are G34, G10, and G9 (Figure 3).

**Figure 4:**
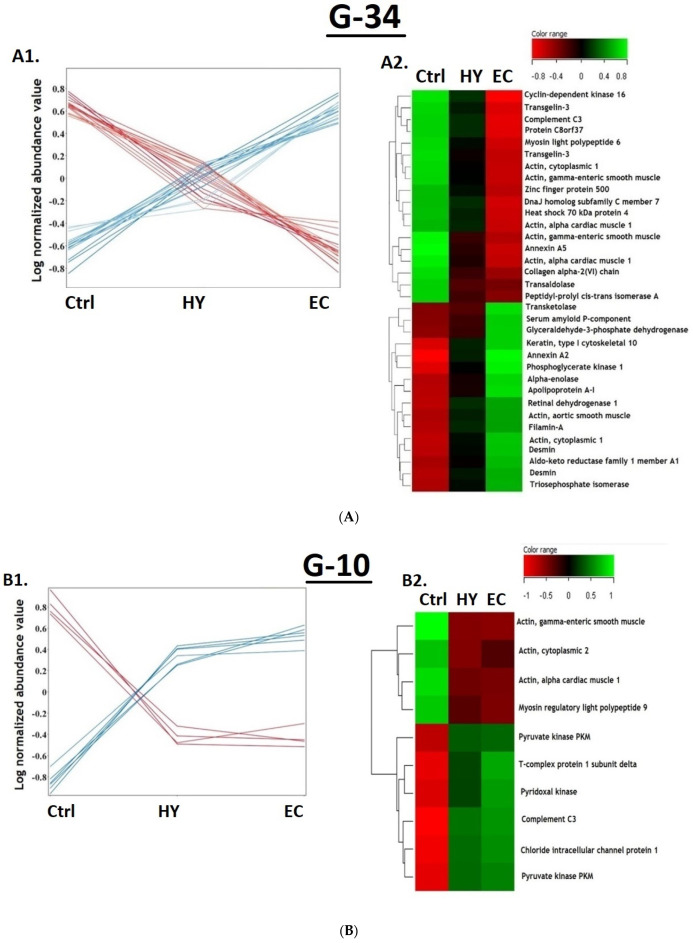

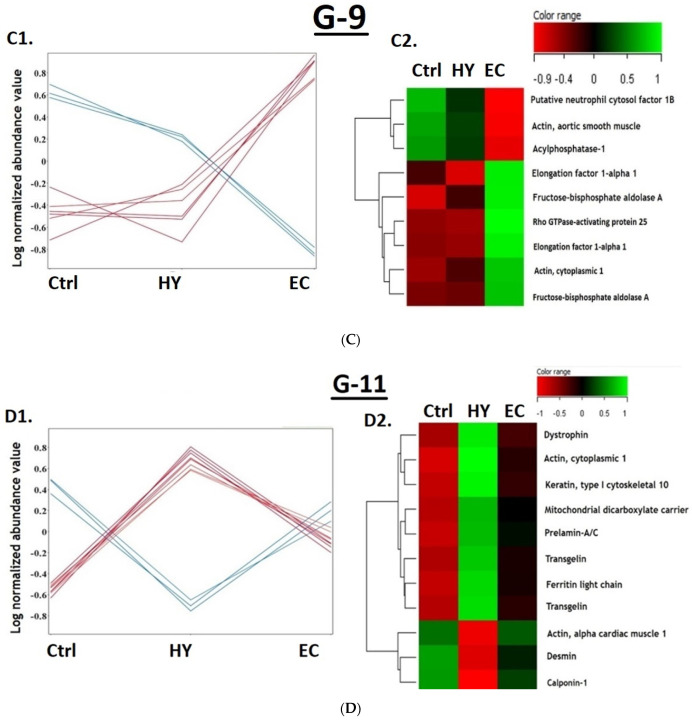

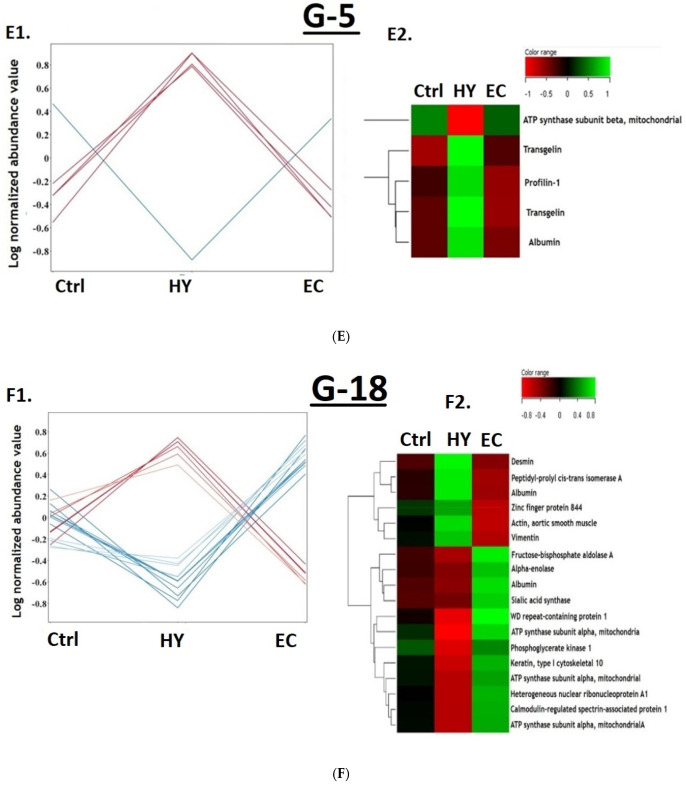
(**A**) Expression profile (**A1**) and heatmap of identified features in subgroup G34 (**A2**). (**B**) Expression profile (**B1**) and heat map of identified features in subgroup G10 (**B2**). (**C**) Expression profile (**C1**) and heatmap of identified features in subgroup G9 (**C2**). (**D**) Expression profile (**D1**) and heat map of identified features in subgroup G11 (**D2**). (**E**) Expression profile (**E1**) and heat map of identified features in subgroup G5 (**E2**). (**F**) Expression profile (**F1**) and heat map of identified features in subgroup G18 (**F2**).

*Subgroup G34:* The G34 panel of protein biomarkers was found to increase or decrease (18 proteins upregulated and 16 proteins downregulated) in terms of expression abundance in a progressive manner from Ctrl to HY and then to EC patients (Figure 4(A1)). The heat map (Figure 4(A2)) clearly represents the proteins in G34 panel with a significant difference between the EC and Ctrl groups, but not between the EC vs. HY and Ctrl vs. HY groups. Therefore, they could possibly be included as candidate biomarkers for the progression of EC from Ctrl and HY.

*Subgroup G10:* Ten proteins were found dysregulated between Ctrl and both HY and EC (Figure 4(A1)). Among G10 proteins, four proteins were downregulated and six proteins were upregulated. The heat map (Figure 4(B2)) represents the identified proteins (*n* = 10) with significant difference between EC and Ctrl but not between EC and HY. This group of proteins (G10) could be considered as a marker for Ctrl patients.

*Subgroup G9:* Nine proteins were differentially expressed in ECs compared to both the HY and Ctrl groups (Figure 4(C1)). Among nine proteins, six were upregulated and three were downregulated. The heat map (Figure 4(C2)) represents the identified proteins with a significant difference between the EC and HY groups, but not between the HY and Ctrl groups. This group of proteins (G9) could be considered as a marker for patients with EC.

### 3.5. The Hyperplasia vs. Control Proteomic Pattern

HY vs. Ctrl comparison shows 26 proteins with statistically significant changes in abundance (*p*-value ≤ 0.05, cutoff ≥ 2.0 fold) as shown in Table 1, Figure 3. Among this 26, the included subgroups are G11, G5, and G10 (Figure 3).

*Subgroup G11***:** Eleven proteins (Figure 4(D1)) were found to have statistically significant differences (three proteins upregulated and eight downregulated) in HY compared to Ctrl. The heatmap (Figure 4(D2)) of the identified proteins shows a significant difference between the HY and EC groups. This group of proteins (G11) could be considered as a candidate marker set for patients with HY.

*Subgroup G5:* The subgroup set G5 showed five proteins with significant differences (one upregulated and four downregulated proteins) in HY compared to both the Ctrl and EC group (Figure 4(E1)). The heatmap (Figure 4(E2)) that represents the identified proteins shows significant difference between HY and Ctrl, but not between the Ctrl and EC groups. Similar to group G11, this group of proteins (G5) could be considered a candidate marker set for patients with HY.

### 3.6. The Cancer vs. Hyperplasia Proteomic Pattern

EC vs. HY comparison shows 32 proteins with statistically significant changes in abundance (*p*-value ≤ 0.05, cutoff ≥ 2.0 fold) as shown in Table 1, Figure 3. Among this 32, the included subgroups are G18, G9, and G5 (Figure 3).

*Subgroup G18:* The expression profile (Figure 4(F1)) represents the identified protein (G18), with a significant difference between EC and HY. The heat map (Figure 4(F2)) represents the identified proteins with a significant difference between the EC and HY groups, but not between the Ctrl groups. This group of proteins (G18) could be considered a candidate marker set for EC patients, similar to G9.

### 3.7. Protein–Protein Interaction Networks

**Figure 5:**
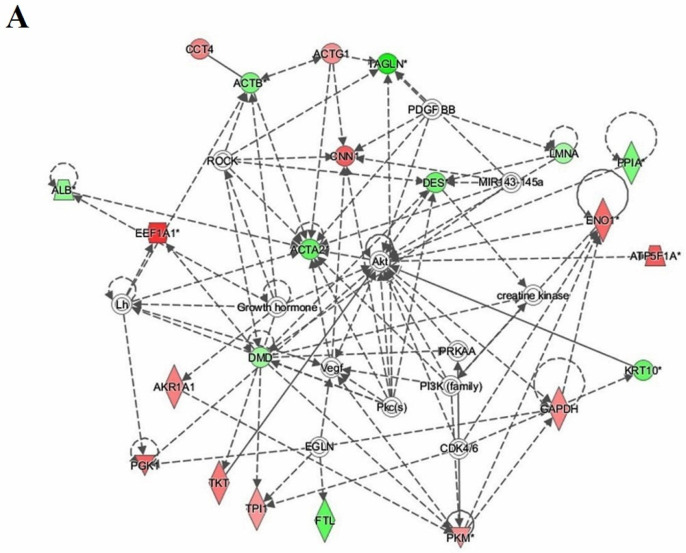

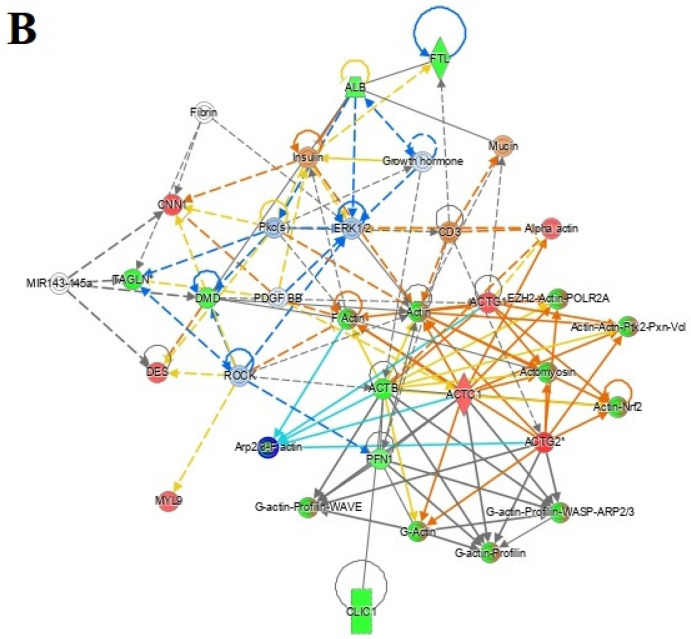

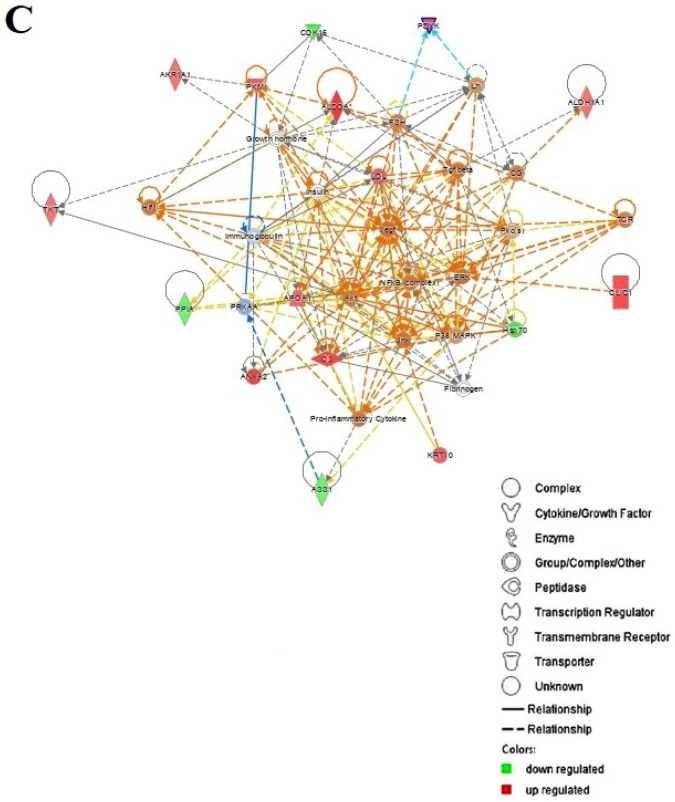
The most enriched interaction network of differentially expressed proteins between the different groups. Red and green nodes indicate up- and downregulated proteins, respectively. Uncolored nodes are proposed by IPA and indicate potential targets that were functionally coordinated with the differentially expressed proteins. Solid lines indicate direct molecular interactions, and dashed lines represent indirect interactions. (**A**) Interaction network of differentially expressed proteins in the EC group compared to the HY group. Central nodes of the pathway related to signaling of ACTA2, AKT, and PI3K were found to be dysregulated between the two states. * appears next to any proteins for which the input file contained more than one identifier (**B**) Interaction network of differentially expressed proteins in the EC group compared to the Ctrl group. Central nodes of the pathway related to signaling of ACTB and ERK1/2 were found to be dysregulated between the two states. (**C**) Interaction network of differentially expressed proteins in the HY group compared to Ctrl group. Central nodes of the pathway related to signaling of VEGF, NFKB, and AKT were found to be dysregulated between the two states.

Bioinformatics analysis using IPA was performed for all 32 proteins that were differentially regulated between the EC and HY states. This study revealed that among the 32 proteins, 22 proteins interacted either directly or indirectly via protein networks (Figure 5). The software computed a score based on the best fit obtained from the input data set of proteins and the biological function database to generate a protein–protein interaction network. The developed network was preferentially enriched for proteins with specific and extensive interactions, in which the interacting proteins were represented as nodes and their biological relationships as a line. Seven interaction networks were identified for proteins that exhibited differential expression profiles based on the data. The highest scoring network (score = 49) (Figure 5) incorporated 22 proteins. The proposed highest interaction network pathway was related to cellular movement, hematological disease, and immunological pathways, with the identified proteins centered around the dysregulation of AKT, actin, aortic smooth muscle (ACTA), and signaling pathways between the two states. Only the top identified pathway is shown (Figure 5A). The canonical pathways enriched in the current data set are shown in Supplementary Figure S3. The canonical pathways shown in Supplementary Figure S3 were sorted into a decreasing log (*p*-value) of enrichment. The three most interesting enriched canonical pathways were glycolysis I, ILK signaling, and actin cytoskeleton signaling. Bioinformatics analysis using IPA was also performed for differentially regulated proteins between EC vs. Ctrl and HY vs. Ctrl states and are shown in Figure 5B (Supplementary Figure S4) and Figure 5C (Supplementary Figure S5), respectively.

The protein analysis through evolutionary relationships (PANTHER) system was used to classify identified proteins according to their molecular function (Supplementary Figure S6A), biological function (Supplementary Figure S6B), and location (Supplementary Figure S6C). The functional category showed that most of the differentially expressed proteins identified had catalytic activity (42%), followed by binding proteins (38%). Considering the biological process, 36% of the identified proteins were involved in the cellular processes, followed by 16% in biological regulation. Location-wise, most of the identified proteins were located in the cellular region (48%), followed by the organellar region (17%).

### 3.8. Multiple Reaction Monitoring (MRM) Mass Spectrometry

**Figure 6:**
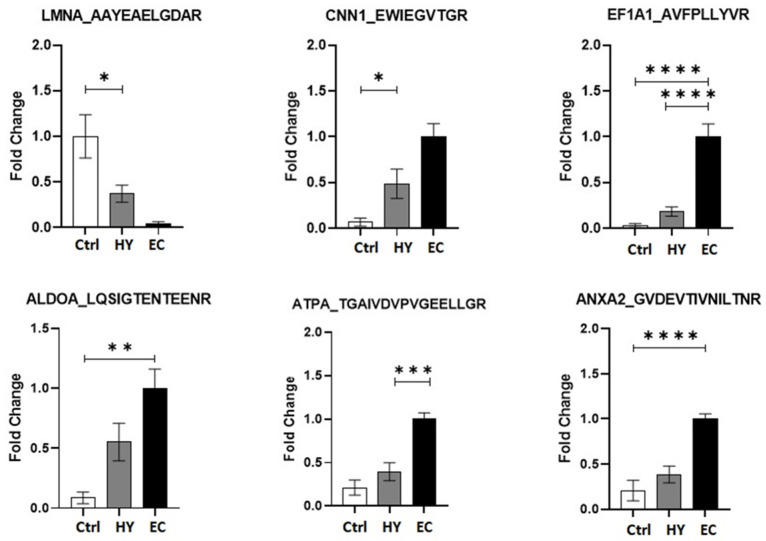
Multiple reaction monitoring (MRM) mass spectrometry for validating study findings. MRM method based on signature peptides was developed to validate the expression of six proteins found in the proteomics approach (2D-DIGE MALDI-TOF-MS). The expression of these six proteins was denoted as fold changes compared among the EC, HY, and Ctrl group. Statistical significance was evaluated using an unpaired *t*-test (*n* = 10), in which * represents *p* ≤ 0.05, ** represents *p* ≤ 0.01, *** represents *p* ≤ 0.001, and **** represents *p* ≤ 0.0001.

Six significantly dysregulated proteins from the 2D-DIGE proteomic profile were selected for validation. Signature peptides for the selected proteins were identified using criteria described previously [26]. Proteins were selected based on their involvement in the protein–protein interaction network pathway. Proteins with a higher number of interactions and fold changes showing both increased and decreased abundance (ANXA2, ALDOA, CNN1, LMNA, EF1A1, and ATPA) were used to confirm the findings. The uniqueness and reliability of these signature peptides were confirmed using Skyline Software V3 and PeptideAtlas [27]. An MRM method was optimized using triple quadrupole mass spectrometry (LC-MS/MS). Representative chromatograms for each protein signature peptide are shown in Supplementary Figure S7 and Table S4. This validation experiment shows that these six selected proteins have similar expression trends compared to the MALDI-TOF results, as shown in Figure 6, with a different fold change value. The expression profiles of these proteins were statistically evaluated using unpaired *t*-tests with PrismPad Software (Dotmatics, Boston, MA, USA).

## 4. Discussion

Several proteomic studies have been conducted to identify EC biomarkers (using plasma, serum, and tissues in their research); none have succeeded in utilizing them for clinical application until now [28,29]. Although these studies were successful in associating a few proteins with EC and the stages of its progression, they were not able to clearly define the relationship between EC and the role of proteins in EC progression. A better understanding of the potential underlying molecular mechanisms is essential to assist in the discovery of more reliable diagnostic and prognostic biomarkers for EC. To the best of our knowledge, this study is the first to employ an untargeted proteomic approach using 2D-DIGE coupled with MALDI-TOF mass spectrometry and bioinformatics analysis to study the proteomic profiles of EC, hyperplasia (HY), and control (Ctrl) groups using tissue samples from Saudi women. Our study found 87 statistically significant abundant proteins of which 53 proteins (28 upregulated and 25 downregulated) were differentially expressed in EC patients compared to Ctrl groups, whereas 26 proteins (8 upregulated and 18 downregulated) were differentially expressed in HY patients compared to Ctrl groups. In HY patients, 32 proteins (19 upregulated and 13 downregulated) were significantly dysregulated compared to EC patients.

### 4.1. Proteins Differentially Expressed in EC vs. Ctrl Patients

Annexin A2 (ANXA2), a 36 kDa membrane protein on the cell surface, belongs to a calcium-regulated phospholipid-binding protein family [30,31]. It is widely expressed in a number of eukaryotic cells, involved in cell survival, and facilitates interactions between intercellular and extracellular microenvironments [30]. Several studies have indicated that ANXA2 is involved in proliferation, angiogenesis, adhesion, invasion, and metastasis [32,33,34]. Aberrant ANXA2 expression is associated with various malignancies, including colorectal cancer [35], pancreatic cancer [36], breast cancer [37], and EC [11]. The data from our study revealed that ANXA2 was overexpressed with a 3-fold change in EC compared to that in the Ctrl group. These data concur with other studies that showed ANXA2 overexpression in pancreatic, breast, and laryngeal cancer tissues [36,37]. In addition, Lorena et al. indicated that overexpression of ANXA2 in ECs can be utilized as a potential biomarker to distinguish between primary endometrial carcinomas and recurrent disease, and that its expression leads to metastasis of ECs [38].

Aldolase (fructose-bisphosphate aldolase A) is a glycolytic enzyme family. It catalyzes the reversible cleavage of fructose-1,6-bisphosphate (FBP) to glyceraldehyde-3-phosphate (GAP) and dihydroxyacetone phosphate (DHAP) [39]. Overexpression of ALDOA protein has been observed in several cancers. Chang et al. indicated that the expression of ALDOA in lung cancer is associated with invasive and metastatic activity [40]. Additionally, ALDOA expression was significantly higher in patients with poorer survival time than in those with better survival [41]. These data indicate that ALDOA expression may be associated with osteosarcoma (OSA) [41]. Hung et al. indicated that ALDOA overexpression in renal cell carcinoma (RCC) was correlated with histological differentiation, metastasis, and prognosis of RCC patients. ALDOA acts as a potential tumor promoter via epithelial-mesenchymal transition (EMT) and the Wnt/β-catenin signaling pathway and may serve as a potential target for diagnosis and therapy [42]. The results from our data revealed that ALDOA has the highest expression among other proteins, with more than a 3-fold change. ALDOA may be used as a potential biomarker to detect and treat patients with EC.

Annexin A5 consists of 319 amino acid residues and has a short unphosphorylated N-terminus compared with other annexins. Annexin A5 has several functions, including signal transduction, cell proliferation and invasion, and anticoagulation [43]. Several studies have shown that the upregulation of annexin A5 is observed in different types of cancers, such as colorectal adenocarcinoma [44], breast cancer [45], and cervical cancer [46], and is capable of promoting tumorigenesis and progression in these cancers. Alternatively, several studies have indicated that annexin A5 is negatively correlated with tumorigenesis in diffuse large B-cell lymphoma and thyroid cancer. This suggests that annexin A5 plays a dual role in cancer cell malignancy and depends on tissue specificity [43]. The protein level of annexin A5 was found to be downregulated in our study, with a 2.7-fold change. It may be negatively correlated with tumorigenesis and progression in patients with EC.

Our data showed that ENO1 was overexpressed with a 2.4-fold change only in EC versus the control group and not in the other groups, and thus could be used as a predictive biomarker for EC. Enolase 1 (ENO1) is a metabolic enzyme implicated in pyruvate synthesis that has been detected in almost all mature tissues. ENO1 acts as a plasminogen receptor, stimulating inflammatory responses in numerous tumors, and is a glycolytic enzyme that catalyzes the penultimate step in glycolysis [46]. It also facilitates the activation of plasmin and degradation of the extracellular matrix [47]. Several glucose transporters and glycolytic enzymes in cancer cells are involved in the Warburg effect, which is capable of activating and overexpressing ENO1. ENO1 has also been found to regulate the development of various cancers, including breast cancer, lung cancer, head and neck cancers, gastric cancer, and glioma [48,49,50]. Yin and his colleagues detected ENO1 overexpression in pancreatic ductal adenocarcinomas (PDAC) patients, and it was positively associated with metastasis of lymph node, clinical stage, and poor prognosis [51]. Another study indicated that the overexpression of ENO1 in bladder cancer (BC) tissue promotes the proliferation and colony formation of BC cells. This implies that ENO1 acts as an oncogene in BC by regulating the cell cycle and apoptosis [52]. Hu et al. demonstrated that the upregulation of ENO1 promotes the growth and metastasis of colorectal cancer (CRC) and is positively associated with poor prognosis. ENO1 may be used as a biological biomarker and therapeutic target [53]. Zhang et al. showed that ENO1 overexpression in SK-BR-3 breast cancer cells and silencing of ENO1 reduced colony formation of these cells through cell cycle arrest and apoptosis [54].

Transgelins were one of the variants of the same protein found at several locations on the gel (Figure S2, Table 1, Supplementary Table S3) in our study. Our data showed Transgelin 3 (TAGL3) to be downregulated in EC versus the Ctrl group, whereas Transgelin (TAGL) was found to be downregulated in HY vs. Ctrl and EC vs. HY groups. Transgelins are found to exist in three isoforms that include transgelin-1 (T-1/SM22α), transgelin-2 (T-2/SM22β), and transgelin-3 (T-3/SM22γ). They are a family of actin-binding proteins involved in altering the structure and morphology of the cytoskeleton. The function of these proteins includes proliferation, migration, and apoptosis in many types of cancers [55]. It has been noticed that transgelins are associated with colorectal cancer (CRC) metastasis. Mo and his colleagues found that these proteins might be implicated in development of early onset CRC, and T-1/SM22α is classified as the top of the biomarker of node status [56]. Another study indicated the upregulation of T-1/SM22α in node-positive CRC in contrast to node-negative disease [57]. On the other hand, several studies indicated that the function of transgelins in cancer is unclear and controversial. These reports showed the loss expression of transgelin T-1/SM22α during progression and tumor suppressor activity of CRC [58,59]. In bladder cancer, several studies demonstrated that high expression of transgelin is associated with poor prognosis, cancer progression, and aggressive pathological features [60,61].

### 4.2. Proteins That Are Differentially Expressed in HY Compared to Ctrl Patients

Calponin is an actin cytoskeleton-binding protein that stabilizes actin filaments. Calponin has three forms, of which calponin 1 (CNN1) plays a vital role in regulating contractility [62]. CNN1, which is generally expressed in smooth muscle cells, can bind to thin filaments of actin, calmodulin, and tropomyosin. It can also inhibit actin-activated myosin Mg-ATPase and Ca^2+^-dependent mobility of actin on immobilized myosin [63]. Thus, CNN1 is believed to play a crucial role in stabilizing actin fibers. In addition, CNN1 has been demonstrated to play a protective role against various cancers. Wand et al. revealed that CNN1 upregulation inhibited breast cancer cell cancerization and might act as a suppressor gene and could be a promising therapeutic target for breast cancer [64]. Takeoka et al. suggested that CNN1 overexpression in human fibrosarcoma cells acts as a tumor suppressor and significantly influences cytoskeletal activities [65]. Mitchell et al. showed that CNN1 is a tumor-suppressive protein that mediates the immune response. It can also be used to distinguish between low- and high-risk ductal carcinoma in situ (DCIS) lesions [66]. Yamane et al. showed that CNN1 expression in mesothelial cells inhibited ovarian cell invasion via a constructed mesothelial cell monolayer. Upregulation of CNN1 in these cells prevents the invasion of cancer cells to a high degree and suppresses ovarian cancer development [67]. Our results showed that CNN1 expression was upregulated by more than a 2.3-fold change in hyperplasia compared to the control group. This protein was not expressed in the EC vs. hyperplasia or EC vs. control groups. This may indicate that the loss of expression of these proteins leads to cancer development. CNN1 could be helpful in disease progression and as a therapeutic target. LMNA (lamin A/C), a protein-coding gene, encodes the lamin A and C isoforms. LMNA is essential for DNA replication, RNA-dependent transcription, and stabilization of the nucleus [68,69].

LMNA expression varies among different types of human malignancies. The high expression of lamin A has been correlated with the development and progression of various cancers, such as ovarian [70], colorectal [71], and prostate cancer [72]. In contrast, other studies have indicated that LMNA expression decreases in endometrial [73], colon [74], and breast [75] cancers. As a result of this reduction, overall survival (OS) is reduced, enhancing tumor recurrence and cancer progression. Cicchillitti et al. suggested that the expression of lamin A is downregulated in ECs of all grades. Lamin A expression is inversely correlated with tumor aggressiveness, and its expression level can be used as a novel biomarker to identify grade 1 EC patients at risk of recurrence [73]. In our study, LMNA protein expression was significantly downregulated in HY vs. control groups, suggesting that LMNA might lead to cancer progression.

### 4.3. Proteins That Are Differentially Expressed in EC Compared to HY Patients

Eukaryotic translation elongation factor 1 alpha (eEF1A) is implicated in the final stages of protein production, and controls cell proliferation and death. It also exhibits chaperone-like activity and modulates the cytoskeleton [76]. The EEF1A pleiotropic protein, overexpressed in human malignancies, including gastric cancer and hepatocellular carcinoma, is associated with poor prognosis [77,78]. Chen et al. proposed that hepatocellular carcinoma patients who overexpressed EEF1A had shorter overall survival and disease-free survival compared to those with low expression. They also indicated that EEF1A overexpression was involved in cancer progression [78]. EEF1A upregulation was also found to be three times higher in gastric cancer tissues than in normal tissues. It is associated with TNM stage, distant metastases, tumor size, and histological type [79]. Pinke et al. revealed that EEF1A is highly overexpressed in primary human ovarian carcinomas and may be used as a prognostic factor [80]. In this study, EEF1A was overexpressed with more than a 2-fold change in EC versus control groups and a 3.11-fold change in EC versus HY groups, but not in HY versus control groups. This suggests that EEF1A may be involved in tumor progression. EEF1A1 could be a novel prognostic biomarker and a promising therapeutic agent for patients with EC.

Mitochondrial ATP synthase consists of multiple subunits of the enzyme complexes. This enzyme is essential for ATP production and functions as a proton-pumping ATPase. ATP synthase is located in the inner mitochondrial membrane [81]. One ATP synthase subunit is ATP synthase F1 subunit alpha (ATP5F1A). Several studies have demonstrated that ATP5F1A is overexpressed in breast carcinoma cell lines with different metastatic potential. They suggested that upregulation of this protein may be implicated in breast cancer progression and metastasis. They are potential biomarkers for diagnosis and prognosis, and therapeutic targets for anti-metastasis and anti-tumor therapy [82,83]. Moreover, ATP5A1 overexpression is associated with tumorigenesis and tumor progression in clear cell renal cell carcinoma [84]. Feichtinger et al. revealed that high ATP5A1 expression has a significant positive correlation with the early onset of prostate cancer [85]. Our data showed that ATP5A was overexpressed by 2.8-fold in EC versus HY groups, and was not detected in other groups.

### 4.4. Interactions of Identified Proteins and Network Connectivity Mapping Using IPA

IPA was used for network pathway analysis to gain insight into the molecular mechanisms of differentially expressed proteins through biological function annotations, protein–protein interaction networks, and the discovery of potential biomarkers. Pathway analysis showed that the cluster of proteins differentially expressed between EC versus Ctrl (Figure 5B) identified dysregulation of the AKT signaling pathway. On the other hand, the network analysis of the endometrial tissue samples from women with HY and Ctrl (Figure 5C) centered around regulation of the actin signaling pathway. A third analysis of EC vs. hyperplasia (Figure 5A), that reflects a continuum of the disease state, showed the involvement of ACTA2 and AKT as well as PI3K signaling pathways. IPA (EC vs. HY) identified the dysregulation of pathways associated with cellular movement, hematological disease, and immunological pathways. Dysregulation in the PI3K/AKT pathway has been observed in various cancers, and is involved in proliferation, apoptosis, invasion, metastasis, tumorigenesis, and drug resistance [86]. Overactivation of PI3K/AKT has been reported to play an essential role in cell growth, survival, and EC pathogenesis [87]. ACTA2 is another central hub in our network, which is known to be involved in maintaining mechanical tension, cell shape, cell migration, and invasion [88]. Aberrant expression of ACTA2 has poor clinical outcomes in different cancers, including breast, lung, and pancreatic cancers [89,90,91].

## 5. Conclusions

A number of biomarker candidates in the blood or tissue for endometrial cancer detection have been reported. Unfortunately, none of these have been translated into routine clinical practice. The panels of protein markers identified in this study could be considered as potential biomarkers of interest for distinguishing between endometrial cancer and hyperplasia and for early diagnosis and progression of endometrial cancer from hyperplasia and normal patients. Further investigations are required towards their utilization as therapeutic targets and prognostic markers for the management of patients with endometrial cancer.

## Figures and Tables

**Table 1:** Proteins identified with changes in abundance between EC vs. Ctrl (A), HY vs. Ctrl (B) and EC vs. HY (C) in endometrial tissue samples. Values for the average ratio between the two states, fold changes, and one-way ANOVA (*p*-value ≤ 0.05) using 2D-DIGE. (Analysis type: MALDI-TOF; database: SwissProt; taxonomy: *Homo sapiens*, EC: endometrial cancer, HY: hyperplasia, Ctrl: controls.)

A: Comparison between EC and Ctrl							
Sl No.:	Spot No. ^a^	Accession No. ^b^	Protein Name	MASCOT ID	*p*-Value ^b^ (ANOVA)	Ratio ^c^ EC/Ctrl	Exp ^d^
1	36	A6NI72	Putative neutrophil cytosol factor 1B	NCF1B_HUMAN	3.74 × 10^−5^	−2.9	DOWN
2	23	P62736	Actin, aortic smooth muscle	ACTA_HUMAN	1.27 × 10^−4^	−2.7	DOWN
3	937	P63267	Actin, gamma-enteric smooth muscle	ACTH_HUMAN	1.84 × 10^−4^	−2.8	DOWN
4	932	P63267	Actin, alpha cardiac muscle 1	ACTC_HUMAN	4.47 × 10^−4^	−2.4	DOWN
5	1218	O00299	Chloride intracellular channel protein 1	CLIC1_HUMAN	7.66 × 10^−4^	2.7	UP
6	58	Q9UI15	Transgelin-3	TAGL3_HUMAN	8.94 × 10^−4^	−2.5	DOWN
7	847	P68104	Elongation factor 1-alpha 1	EF1A1_HUMAN	9.87 × 10^−4^	2.2	UP
8	852	P42331	Rho GTPase-activating protein 25	RHG25_HUMAN	0.001	2.7	UP
9	1099	P07355	Annexin A2	ANXA2_HUMAN	0.001	3.0	UP
10	626	P01024	Complement C3	CO3_HUMAN	0.002	2.9	UP
11	290	Q00536	Cyclin-dependent kinase 16	CDK16_HUMAN	0.002	−2.6	DOWN
12	244	Q9UI15	Transgelin-3	TAGL3_HUMAN	0.002	−2.9	DOWN
13	865	P68104	Elongation factor 1-alpha 1	EF1A1_HUMAN	0.002	2.6	UP
14	1347	P63261	Actin, cytoplasmic 2	ACTG_HUMAN	0.002	−2.0	DOWN
15	993	P04075	Fructose-bisphosphate aldolase A	ALDOA_HUMAN	0.002	3.1	UP
16	1117	P08758	Annexin A5	ANXA5_HUMAN	0.002	−2.7	DOWN
17	729	P50991	T-complex protein 1 subunit delta	TCPD_HUMAN	0.002	2.8	UP
18	291	P34932	Heat shock 70 kDa protein 4	HSP74_HUMAN	0.002	−2.4	DOWN
19	1593	Q01995	Transgelin	TAGL_HUMAN	0.003	−2	DOWN
20	1647	P60709	Actin, cytoplasmic 1	ACTB_HUMAN	0.003	2.80	UP
21	962	P60709	Actin, cytoplasmic 1	ACTB_HUMAN	0.003	2.41	UP
22	215	Q9UI15	Transgelin-3	TAGL3_HUMAN	0.003	−2.60	DOWN
23	320	Q96NL8	Protein C8orf37	CH037_HUMAN	0.004	−2.62	DOWN
24	1697	P60660	Myosin light polypeptide 6	MYL6_HUMAN	0.004	−2.55	DOWN
25	279	Q99615	DnaJ homolog subfamily C member 7	DNJC7_HUMAN	0.004	−2.41	DOWN
26	947	P63267	Actin, gamma-enteric smooth muscle	ACTH_HUMAN	0.004	−2.54	DOWN
27	207	P60709	Actin, cytoplasmic 1	ACTB_HUMAN	0.005	2.49	UP
28	1692	P24844	Myosin regulatory light polypeptide 9	MYL9_HUMAN	0.005	−2.34	DOWN
29	950	P13645	Keratin, type I cytoskeletal 10	K1C10_HUMAN	0.006	2.56	UP
30	935	P68032	Actin, alpha cardiac muscle 1	ACTC_HUMAN	0.006	−2.58	DOWN
31	761	P14618	Pyruvate kinase PKM	KPYM_HUMAN	0.008	2.13	UP
32	307	P63267	Actin, alpha cardiac muscle 1	ACTC_HUMAN	0.009	−2.32	DOWN
33	785	P62736	Actin, aortic smooth muscle	ACTA_HUMAN	0.009	−2.09	DOWN
34	723	P14618	Pyruvate kinase PKM	KPYM_HUMAN	0.009	2.55	UP
35	209	O60304	Zinc finger protein 500	ZN500_HUMAN	0.01	−2.28	DOWN
36	901	P63267	Actin, gamma-enteric smooth muscle	ACTH_HUMAN	0.011	−2.47	DOWN
37	534	P17661	Desmin	DESM_HUMAN	0.012	2.17	UP
38	982	O00764	Pyridoxal kinase	PDXK_HUMAN	0.012	2.65	UP
39	843	P06733	Alpha-enolase	ENOA_HUMAN	0.016	2.37	UP
40	1106	P02647	Apolipoprotein A-I	APOA1_HUMAN	0.02	2.44	UP
41	208	P12110	Collagen alpha-2(VI) chain	CO6A2_HUMAN	0.022	−2.26	DOWN
42	998	P04075	Fructose-bisphosphate aldolase A	ALDOA_HUMAN	0.022	2.22	UP
43	1270	P60174	Triosephosphate isomerase	TPIS_HUMAN	0.024	2.16	UP
44	775	P00352	Retinal dehydrogenase 1	AL1A1_HUMAN	0.027	2.18	UP
45	1043	P14550	Aldo-keto reductase family 1 member A1	AK1A1_HUMAN	0.029	2.18	UP
46	977	P60709	Actin, cytoplasmic 1	ACTB_HUMAN	0.032	2.36	UP
47	607	P29401	Transketolase	TKT_HUMAN	0.034	2.17	UP
48	1067	P17661	Desmin	DESM_HUMAN	0.038	2.10	UP
49	313	P00966	Argininosuccinate synthase	ASSY_HUMAN	0.04	−2.03	DOWN
50	896	P17661	Desmin	DESM_HUMAN	0.04	2.31	UP
51	231	P62937	Peptidyl-prolyl cis-trans isomerase A	PPIA_HUMAN	0.042	−2.12	DOWN
52	905	P04406	Glyceraldehyde-3-phosphate dehydrogenase	G3P_HUMAN	0.042	2.15	UP
53	1098	P21333	Filamin-A	FLNA_HUMAN	0.043	2.12	UP
**B: Comparison between HY and Ctrl**							
**Sl No.:**	**Spot No. ^a^**	**Accession No. ^b^**	**Protein Name**	**MASCOT ID**	***p*-Value ^b^** **(ANOVA)**	**Ratio ^c^ HY/Ctrl**	**Exp ^d^**
1	937	P63267	Actin, gamma-enteric smooth muscle	ACTH_HUMAN	1.84 × 10^−4^	2.74	UP
2	932	P63267	Actin, alpha cardiac muscle 1	ACTC_HUMAN	4.47 × 10^−4^	2.37	UP
3	1218	O00299	Chloride intracellular channel protein 1	CLIC1_HUMAN	7.66 × 10^−4^	−2.48	DOWN
4	1677	P02792	Ferritin light chain	FRIL_HUMAN	0.001	−2.39	DOWN
5	1645	Q01995	Transgelin	TAGL_HUMAN	0.001	−2.22	DOWN
6	767	P06576	ATP synthase subunit beta, mitochondrial	ATPB_HUMAN	0.002	2.53	UP
7	626	P01024	Complement C3	CO3_HUMAN	0.002	−2.62	DOWN
8	1347	P63261	Actin, cytoplasmic 2	ACTG_HUMAN	0.002	2.32	UP
9	729	P50991	T-complex protein 1 subunit delta	TCPD_HUMAN	0.002	−2.19	DOWN
10	1647	P60709	Actin, cytoplasmic 1	ACTB_HUMAN	0.003	−2.71	DOWN
11	1644	Q01995	Transgelin	TAGL_HUMAN	0.003	−2.34	DOWN
12	1639	P13645	Keratin, type I cytoskeletal 10	K1C10_HUMAN	0.004	−2.54	DOWN
13	1692	P24844	Myosin regulatory light polypeptide 9	MYL9_HUMAN	0.005	2.12	UP
14	646	P17661	Desmin	DESM_HUMAN	0.007	2.21	UP
15	1136	P51911	Calponin-1	CNN1_HUMAN	0.008	2.37	UP
16	761	P14618	Pyruvate kinase PKM	KPYM_HUMAN	0.008	−2.06	DOWN
17	723	P14618	Pyruvate kinase PKM	KPYM_HUMAN	0.009	−2.40	DOWN
18	1636	Q01995	Transgelin	TAGL_HUMAN	0.01	−2.74	DOWN
19	982	O00764	Pyridoxal kinase	PDXK_HUMAN	0.012	−2.10	DOWN
20	381	P02768	Albumin	ALBU_HUMAN	0.015	−2.19	DOWN
21	486	Q9UBX3	Mitochondrial dicarboxylate carrier	DIC_HUMAN	0.018	−2.17	DOWN
22	695	P68032	Actin, alpha cardiac muscle 1	ACTC_HUMAN	0.023	2.09	UP
23	1732	P07737	Profilin-1	PROF1_HUMAN	0.024	−2.01	DOWN
24	369	P02545	Prelamin-A/C	LMNA_HUMAN	0.026	−2.26	DOWN
25	1594	Q01995	Transgelin	TAGL_HUMAN	0.03	−2.35	DOWN
26	1599	P11532	Dystrophin	DMD_HUMAN	0.042	−2.36	DOWN
**C: Comparison between EC and HY**							
**Sl No.:**	**Spot No. ^a^**	**Accession No. ^b^**	**Protein Name**	**MASCOT ID**	***p*-Value ^b^** **(ANOVA)**	**Ratio ^c^ EC/HY**	**Exp ^d^**
1	36	A6NI72	Putative neutrophil cytosol factor 1B	NCF1B_HUMAN	3.74 × 10^−5^	−2.06	DOWN
2	23	P62736	Actin, aortic smooth muscle	ACTA_HUMAN	1.27 × 10^−4^	−2.11	DOWN
3	54	P07311	Acylphosphatase-1	ACYP1_HUMAN	3.57 × 10^−4^	−2.00	DOWN
4	847	P68104	Elongation factor 1-alpha 1	EF1A1_HUMAN	9.87 × 10^−4^	3.11	UP
5	852	P42331	Rho GTPase-activating protein 25	RHG25_HUMAN	0.001	2.81	UP
6	767	P06576	ATP synthase subunit beta, mitochondrial	ATPB_HUMAN	0.002	2.32	UP
7	1613	P62736	Actin, aortic smooth muscle	ACTA_HUMAN	0.002	−2.43	DOWN
8	865	P68104	Elongation factor 1-alpha 1	EF1A1_HUMAN	0.002	2.66	UP
9	993	P04075	Fructose-bisphosphate aldolase A	ALDOA_HUMAN	0.002	2.17	UP
10	1103	P09651	Heterogeneous nuclear ribonucleoprotein A1	ROA1_HUMAN	0.003	2.19	UP
11	962	P60709	Actin, cytoplasmic 1	ACTB_HUMAN	0.003	2.01	UP
12	1644	Q01995	Transgelin	TAGL_HUMAN	0.003	−2.66	DOWN
13	638	O75083	WD repeat-containing protein 1	WDR1_HUMAN	0.004	2.9	UP
14	1679	P17661	Desmin	DESM_HUMAN	0.007	−2.26	DOWN
15	696	P02768	Albumin	ALBU_HUMAN	0.007	2.18	UP
16	1636	Q01995	Transgelin	TAGL_HUMAN	0.01	−2.26	DOWN
17	711	P25705	ATP synthase subunit alpha, mitochondria	ATPA_HUMAN	0.01	2.79	UP
18	899	P04075	Fructose-bisphosphate aldolase A	ALDOA_HUMAN	0.011	2.41	UP
19	1604	P62937	Peptidyl-prolyl cis-trans isomerase A	PPIA_HUMAN	0.013	−2.34	DOWN
20	381	P02768	Albumin	ALBU_HUMAN	0.015	−2.34	DOWN
21	1283	P13645	Keratin, type I cytoskeletal 10	K1C10_HUMAN	0.021	2.29	UP
22	1083	Q5T5Y3	Calmodulin-regulated spectrin-associated protein 1	CAMP1_HUMAN	0.022	2.16	UP
23	998	P04075	Fructose-bisphosphate aldolase A	ALDOA_HUMAN	0.022	2.14	UP
24	1732	P07737	Profilin-1	PROF1_HUMAN	0.024	−2.4	DOWN
25	1030	Q9NR45	Sialic acid synthase	SIAS_HUMAN	0.026	2.00	UP
26	888	P08670	Vimentin	VIME_HUMAN	0.027	−2.26	DOWN
27	870	P00558	Phosphoglycerate kinase 1	PGK1_HUMAN	0.028	2.20	UP
28	447	P02768	Albumin	ALBU_HUMAN	0.029	−2.33	DOWN
29	732	P25705	ATP synthase subunit alpha, mitochondrial	ATPA_HUMAN	0.036	2.14	UP
30	930	P06733	Alpha-enolase	ENOA_HUMAN	0.037	2.03	UP
31	1618	Q08AG5	Zinc finger protein 844	ZN844_HUMAN	0.039	−2.15	DOWN
32	1046	P25705	ATP synthase subunit alpha, mitochondrial	ATPA_HUMAN	0.046	2.11	UP

^a^ Protein accession number for SWISSPROT database. ^b^ *p*-Value (ANOVA). ^c^ Ratio between the groups. ^d^ Protein expression between the groups.

## Data Availability

All data generated or analyzed in the current study are included in this article.

## Supplementary Materials

The following supporting information can be downloaded at https://www.mdpi.com/article/10.3390/cells11132119/s1, Figure S1: Representative overlap of Cy3/Cy5 images; Figure S2: Representative image of protein spots from the endometrial tissue from the study groups; Figure S3: The diagram shows the top canonical pathway; Figure S4: Top canonical pathways ranked by the *p*-values obtained using Ingenuity Pathway Analysis (IPA) performed for all differentially regulated proteins between EC and Ctrl states; Figure S5: Top canonical pathways ranked by the *p*-values obtained using Ingenuity Pathway Analysis (IPA) performed for all differentially regulated proteins between HY and Ctrl states; Figure S6: Comparative depiction (%) of identified proteins categorized into groups; Figure S7: Representative extracted ion chromatograms for the selected protein; Figure S8: Western blot assay of the chosen proteins identified by 2D-DIGE analysis; Table S1: Clinico-pathological characteristics; Table S2: Experimental design; Table S3: Mass spectrometry list of significant differentially abundant proteins; Table S4: Experimental conditions of the selected protein signature peptides used for proteomics validation.
