# Supplementary material for: Tissue-Based Proteomic Profiling in Patients with Hyperplasia and Endometrial Cancer
Source: Cells. 2022 Jul 5;11(13):2119. doi: 10.3390/cells11132119 (PMC9265283; doi:10.3390/cells11132119)
Supplement: Supplementary file 1 [file cells-11-02119-s001.zip › cells-1726385-supplementary.pdf]

Supplementary Data:

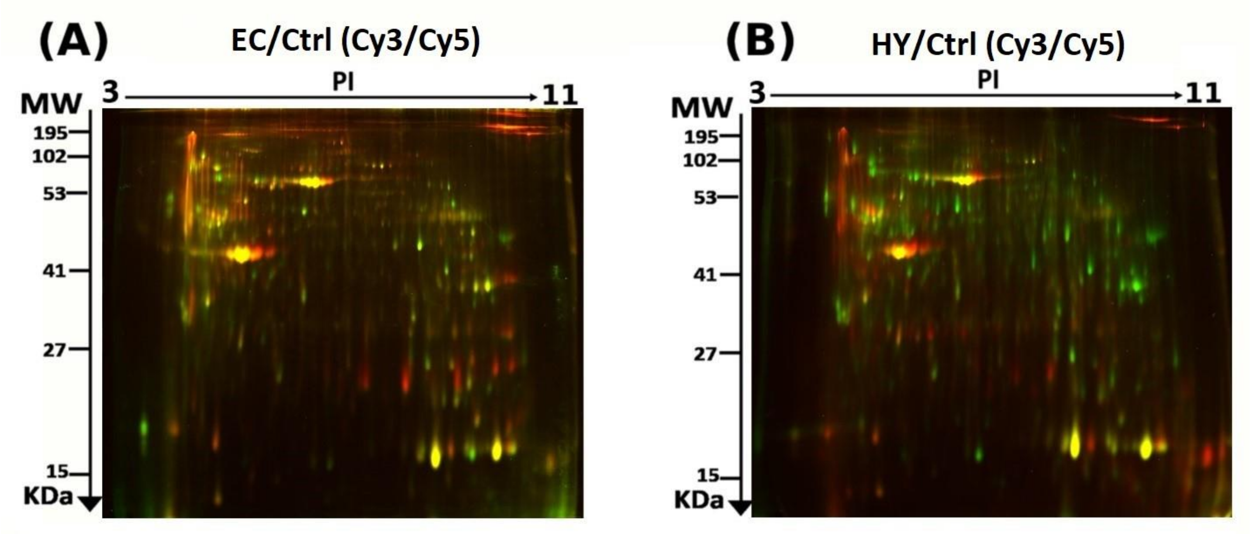

Figure S1. Representative overlay of Cy3(Green)/Cy5(Red) images of (**A**) EC/Crtl; (**B**) representative overlay of Cy3(Green)/Cy5(Red) images of HY/Ctrl, respectively.

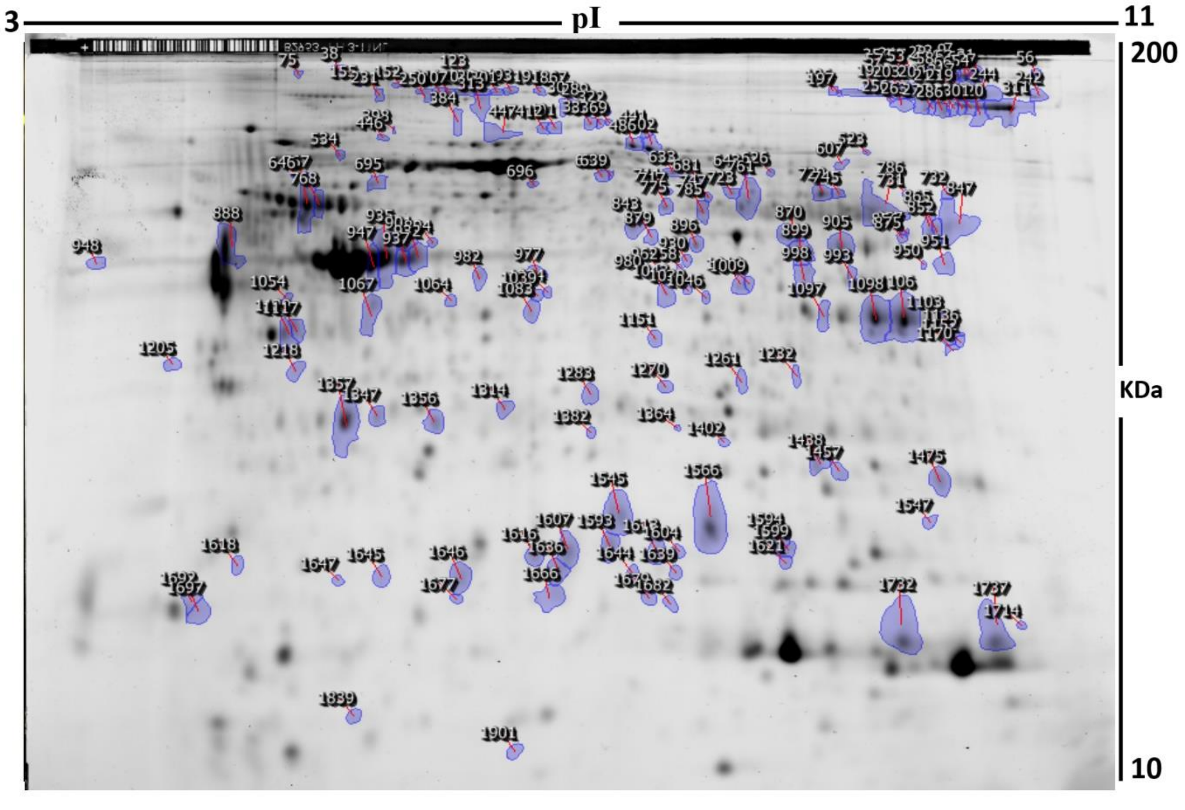

Figure S2: Representative image of statistically significant differential expression (ANOVA $P \leq 0.05$) protein spots (numbered spots) between EC, HY, and Ctrl in the human endometrial tissues.

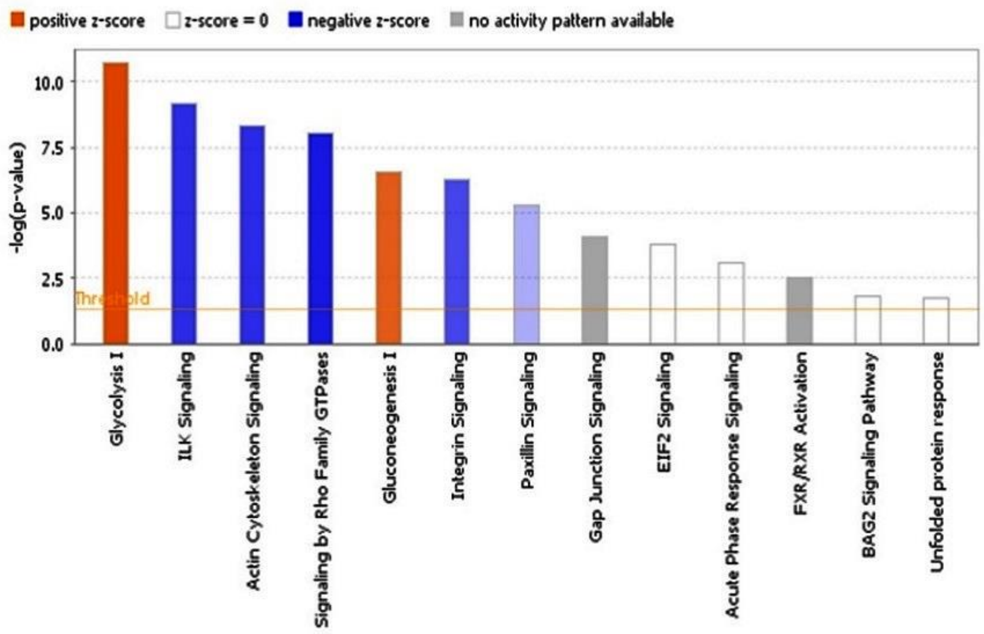

Figure S3. The diagram shows the top canonical pathways ranked by the *P*-values obtained by the Ingenuity Pathway Analysis (IPA) performed for all the 32 differentially regulated proteins between EC and HY states.

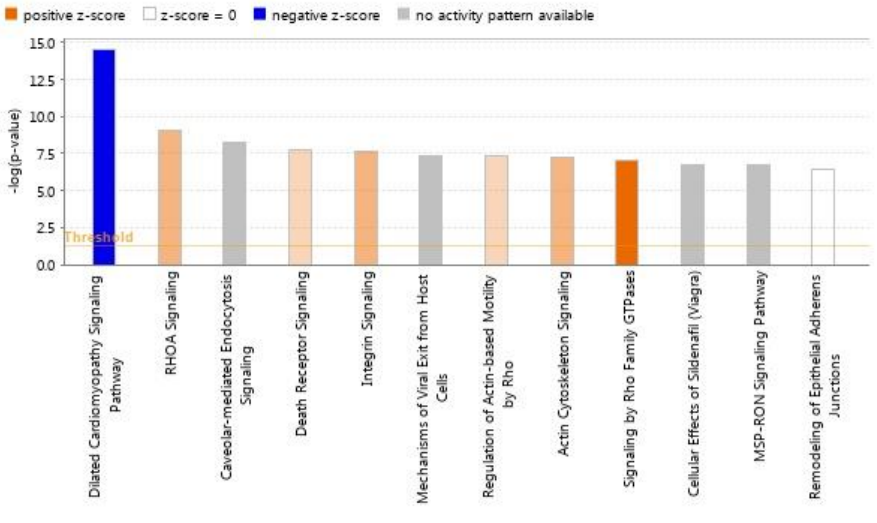

Figure S4. Top canonical pathways ranked by the P-values obtained using Ingenuity Pathway Analysis (IPA) performed for all differentially regulated proteins between EC and Ctrl states.

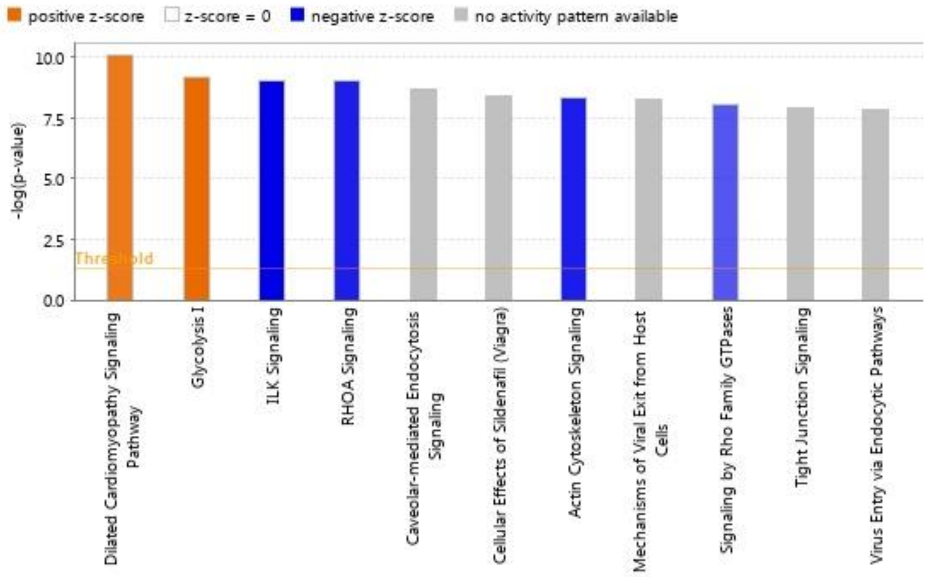

Figure S5. Top canonical pathways ranked by the P-values obtained using Ingenuity Pathway Analysis (IPA) performed for all differentially regulated proteins between HY and Ctrl states.

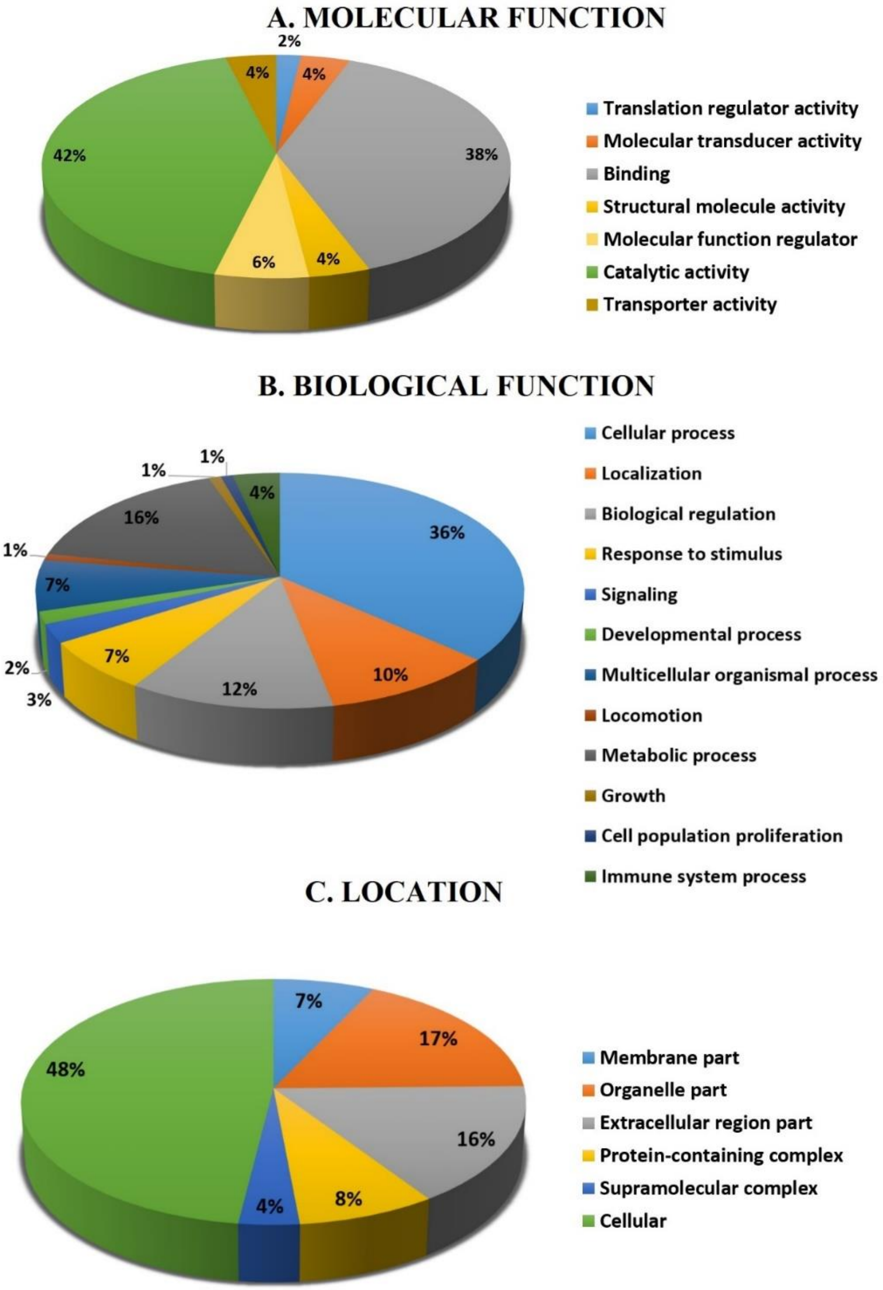

Figure S6. Comparative depiction (%) of identified proteins categorized into groups according to their Molecular Function (**A**), Biological Function (**B**), and Location (**C**).

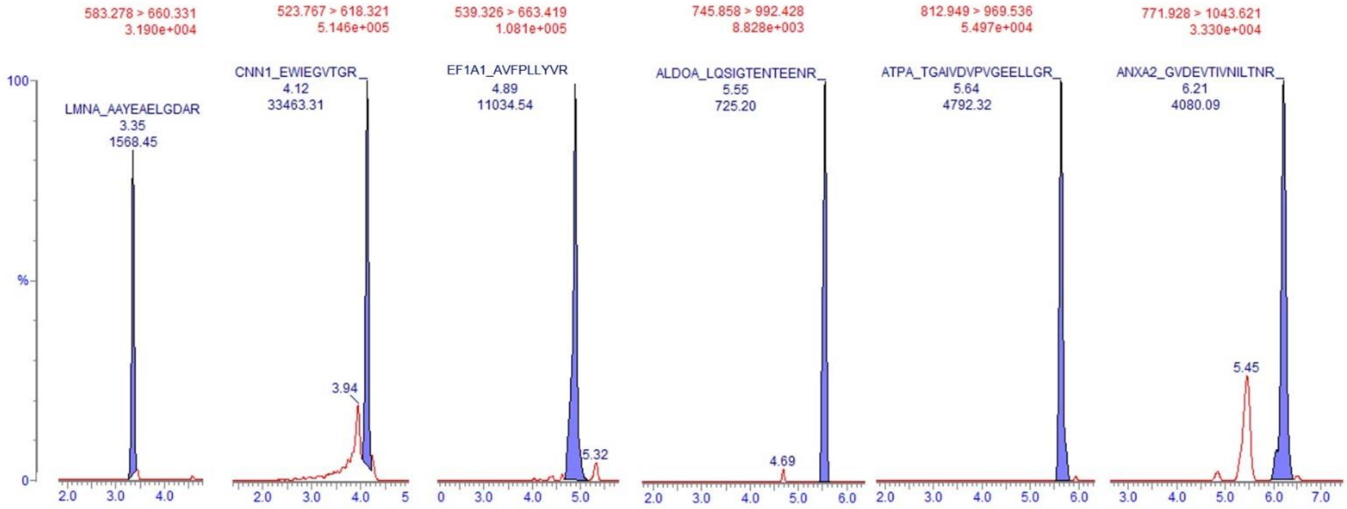

Figure S7. Representative extracted ion chromatograms for the selected proteins' signature peptides based on the multiple-reaction monitoring method developed for validation.

Confirmation of changes in Selected Proteins by Immunoblotting

Immunoblotting assay was performed in the current study to further confirm the findings. Four proteins with statistically significant differential abundance were chosen and determined by immunoblotting. Primary monoclonal antibodies against Alpha-enolase (ENOA), Glyceraldehyde-3-phosphate dehydrogenase (G3P), Annexin A5 (ANXA5), Apolipoprotein A-I (APOA1), and beta actin (β-actin) were used. An equal amount of protein (5 μg) from each sample from the three groups (EC = 10, HY = 10, and EC = 10, i.e. 50 μg from each group) was taken and pooled together; one-dimensional discontinuous slab gel electrophoresis (12% sodium dodecyl sulfate (SDS)-polyacrylamide gel) was used to separate. A mini trans-blot electrotransfer cell (BioRad, California, CA, USA) was employed to transfer proteins from the run gels to an Immobilon-P, polyvinylidene difluoride (PVDF) transfer membrane (Millipore, Massachusetts, MA, USA). To test the efficiency of the transfer, the membranes were stained with Ponceau-S. Subsequently, the membranes were blocked with tris-buffered saline (TBS) containing 5% fat-free milk (FFM), for one hour at room temperature, and then the membranes were rinsed three times with TBS-T in 10 mM Tris–HCl, 150 mM NaCl, 0.1% Tween 20 buffer. After rinsing, the membranes were incubated with the selected primary antibodies at dilution of (1:200) using a blocking buffer. Membranes were then incubated with the matched immunoglobulin G (IgG)-horseradish peroxidase (HRP)-conjugated secondary antibody, and the enhanced chemiluminescence (ECL, Thermo Fisher Scientific, Massachusetts, MA, USA) was used to detect the immunoreactive bands. These bands were visualized by scanning with Sapphire Biomolecular Imager (Azure Bio systems, Dublin, OH, USA) and digitalized via the image analysis software Sapphire Capture system (Azure Biosystems, Dublin, OH, USA).

Immunoblot assay confirmed the expression of the selected proteins (Figure S8). The proteins selected for confirmation were ENOA, G3P, ANXA5, and APOA1. Immunoblots revealed that the endometrial tissue protein expression of G3P, APOA1, and ENOA1 was upregulated in EC compared to HY and Ctrl. ANXA5 was found to be downregulated in EC compared to HY and Ctrl. Immunoblot data were normalized to the housekeeping protein β-actin. Similar results for expression of proteins were found with the 2D-DIGE experiment.

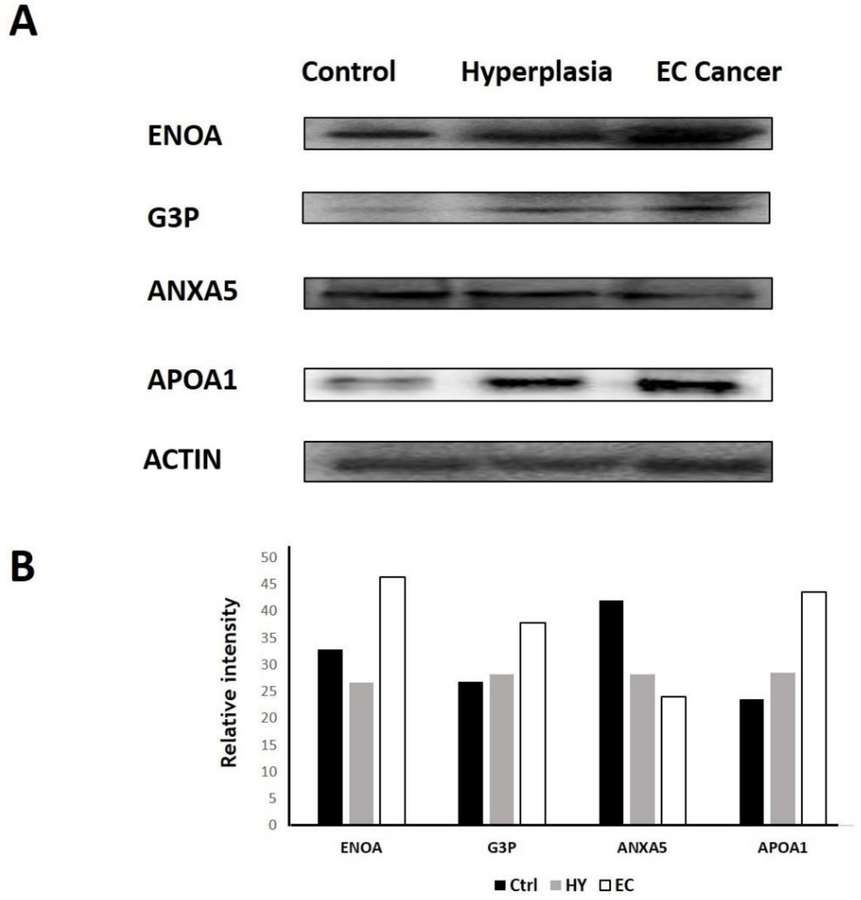

Figure S8. Western blot assay of the chosen proteins identified by 2D-DIGE analysis. Samples from Ctrl, EC, and HY groups were pooled together and used for Western blot analysis. The results of Western blot study were similar to the findings of 2D-DIGE (**A**). Graphical analysis of the relative expression data of normalized protein blots between the Ctrl, HY, and EC is shown. The presented data are shown as histograms of the mean ± SD (**B**).

Table S1. Clinicopathological characteristics of the 36 female patients enrolled in the study

Sample ID	Sample type	Age	Diagnosis	Stage/Grade
EC1	Cancer	71	Endometrioid endometrial adenocarcinoma	1A/G2
EC2	Cancer	74	Endometrioid endometrial adenocarcinoma	1A/G2
EC 3	Cancer	76	Endometrioid endometrial adenocarcinoma	1A/G1
EC 4	Cancer	61	Endometrioid endometrial adenocarcinoma	1B/G3
EC 5	Cancer	56	Endometrioid endometrial adenocarcinoma	1B/G1
EC 6	Cancer	50	Endometrioid endometrial adenocarcinoma	1A/G1
EC 7	Cancer	46	Endometrioid endometrial adenocarcinoma	3A/GA
EC 8	Cancer	70	Endometrioid endometrial adenocarcinoma	3C/G3
EC 9	Cancer	69	Endometrioid endometrial adenocarcinoma	3C/G2
EC 10	Cancer	51	Endometrioid endometrial adenocarcinoma	3A/G2
EC 11	Cancer	74	Endometrioid endometrial adenocarcinoma	1A/G3
EC 12	Cancer	61	Endometrioid endometrial adenocarcinoma	1A/G2
HY1	Hyperplasia	55	hyperplastic endometrium without atypia	
HY2	Hyperplasia	58	hyperplastic endometrium with atypia	
HY3	Hyperplasia	53	hyperplastic endometrium with atypia	
HY4	Hyperplasia	61	hyperplastic endometrium with atypia	
HY5	Hyperplasia	67	hyperplastic endometrium without atypia	
HY6	Hyperplasia	60	hyperplastic endometrium with atypia	
HY7	Hyperplasia	67	hyperplastic endometrium with atypia	
HY8	Hyperplasia	49	hyperplastic endometrium with atypia	
HY9	Hyperplasia	62	hyperplastic endometrium with atypia	
HY10	Hyperplasia	60	hyperplastic endometrium with atypia	
HY11	Hyperplasia	67	hyperplastic endometrium with atypia	
HY12	Hyperplasia	61	hyperplastic endometrium with atypia	
Ctrl1	Control	52	Normal endometrium	
Ctrl 2	Control	59	Normal endometrium	
Ctrl 3	Control	63	Normal endometrium	
Ctrl 4	Control	61	Normal endometrium	
Ctrl 5	Control	63	Normal endometrium	
Ctrl 6	Control	57	Normal endometrium	
Ctrl 7	Control	62	Normal endometrium	
Ctrl8	Control	56	Normal endometrium	
Ctrl 9	Control	60	Normal endometrium	
Ctrl 10	Control	58	Normal endometrium	
Ctrl 11	Control	50	Normal endometrium	
Ctrl 12	Control	56	Normal endometrium	

Table S2. Experimental design: 36 samples run on 18 2D-PAGE gels, samples were labeled randomly with Cy3 and Cy5, and a pooled sample was used as an internal standard and was stained with Cy2 (EC: cancer, HY: hyperplasia, Ctrl: controls).

**Gel**	**Cy3**	**Cy5**	**Cy2**
1	HY1	EC1	Pooled sample
2	Ctrl1	HY2	Pooled sample
3	EC2	Ctrl2	Pooled sample
4	HY3	EC3	Pooled sample
5	Ctrl3	HY4	Pooled sample
6	EC4	Ctrl4	Pooled sample
7	HY5	EC5	Pooled sample
8	Ctrl5	HY6	Pooled sample
9	EC6	Ctrl6	Pooled sample
10	HY7	EC7	Pooled sample
11	Ctrl7	HY8	Pooled sample
12	EC8	Ctrl8	Pooled sample
13	HY9	EC9	Pooled sample
14	Ctrl9	HY10	Pooled sample
15	EC10	Ctrl10	Pooled sample
16	HY11	EC11	Pooled sample
17	Ctrl11	HY12	Pooled sample
18	EC12	Ctrl12	Pooled sample

Table S3. Mass spectrometry list of significant differentially abundant proteins between EC, HY, and Ctrl states identified in endometrial tissue samples, using 2D-DIGE MALDI-TOF. Protein name, accession number, Mascot score, MS% coverage, protein MW, and pI values according to Uniprot database are listed.

Sl no:	Spot No[a]	Accession No[b]	Protein Name	MASCOT ID	Pi[c]	MW[d]	Cov%	Score [e]	Matching peptides
1	36	A6NI72	Putative neutrophil cytosol factor 1B	NCF1B_HUMAN	9.20	45017	30	57	8
2	23	P62736	Actin, aortic smooth muscle	ACTA_HUMAN	5.23	42381	31	90	12
3	937	P63267	Actin, gamma-enteric smooth muscle	ACTH_HUMAN	5.31	42108	62	155	20
4	54	P07311	Acylphosphatase-1	ACYP1_HUMAN	9.30	11954	39	57	7
5	932	P63267	Actin, alpha cardiac muscle 1	ACTC_HUMAN	5.23	42334	52	195	22
6	1218	O00299	Chloride intracellular channel protein 1	CLIC1_HUMAN	5.09	27248	52	130	12
7	58	Q9UI15	Transgelin-3	TAGL3_HUMAN	6.84	22629	66	62	10
8	847	P68104	Elongation factor 1-alpha 1	EF1A1_HUMAN	9.10	50457	42	71	18
9	852	P42331	Rho GTPase-activating protein 25	RHG25_HUMAN	6.04	73902	15	58	6
10	1677	P02792	Ferritin light chain	FRIL_HUMAN	5.51	20064	61	113	13
11	1099	P07355	Annexin A2	ANXA2_HUMAN	7.57	38808	72	181	28
12	1645	Q01995	Transgelin	TAGL_HUMAN	8.87	22653	35	66	10
13	767	P06576	ATP synthase subunit beta, mitochondrial	ATPB_HUMAN	5.26	56525	59	128	35
14	626	P01024	Complement C3	CO3_HUMAN	6.02	188569	34	158	52
15	290	Q00536	Cyclin-dependent kinase 16	CDK16_HUMAN	7.23	55909	23	63	10
16	244	Q9UI15	Transgelin-3	TAGL3_HUMAN	6.84	22629	66	65	11
17	1613	P62736	Actin, aortic smooth muscle	ACTA_HUMAN	5.23	42381	42	82	14
18	865	P68104	Elongation factor 1-alpha 1	EF1A1_HUMAN	9.10	50451	32	76	13
19	1347	P63261	Actin, cytoplasmic 2	ACTG_HUMAN	5.31	42108	33	79	14
20	993	P04075	Fructose-bisphosphate aldolase A	ALDOA_HUMAN	8.30	39851	81	192	25
21	1117	P08758	Annexin A5	ANXA5_HUMAN	4394	35971	45	128	14

22	729	P50991	T-complex protein 1 subunit delta	TCPD_HUMAN	7.96	58401	36	94	21
23	291	P34932	Heat shock 70 kDa protein 4	HSP74_HUMAN	5.11	95127	29	57	20
24	1103	P09651	Heterogeneous nuclear ribonucleoprotein A1	ROA1_HUMAN	9.17	38837	45	94	17
25	1593	Q01995	Transgelin	TAGL_HUMAN	8.87	2268	56	91	16
26	1647	P60709	Actin, cytoplasmic 1	ACTB_HUMAN	5.29	42052	32	88	12
27	962	P60709	Actin, cytoplasmic 1	ACTB_HUMAN	5.29	42052	45	159	20
28	215	Q9UI15	Transgelin-3	TAGL3_HUMAN	6.84	22629	72	66	14
29	1644	Q01995	Transgelin	TAGL_HUMAN	8.87	22653	62	107	24
30	1639	P13645	Keratin, type I cytoskeletal 10	K1C10_HUMAN	5.13	59020	39	116	26
31	320	Q96NL8	Protein C8orf37	CH037_HUMAN	6.52	23993	55	58	15
32	1697	P60660	Myosin light polypeptide 6	MYL6_HUMAN	4.56	17090	70	76	13
33	638	O75083	WD repeat-containing protein 1	WDR1_HUMAN	6.17	66836	25	74	14
34	279	Q99615	DnaJ homolog subfamily C member 7	DNJC7_HUMAN	6.56	57203	18	62	10
35	947	P63267	Actin, gamma-enteric smooth muscle	ACTH_HUMAN	5.23	42334	75	183	32
36	207	P60709	Actin, cytoplasmic 1	ACTB_HUMAN	5.29	42052	41	104	21
37	1692	P24844	Myosin regulatory light polypeptide 9	MYL9_HUMAN	4.80	19871	52	70	12
38	950	P13645	Keratin, type I cytoskeletal 10	K1C10_HUMAN	5.13	59020	34	74	19
39	935	P68032	Actin, alpha cardiac muscle 1	ACTC_HUMAN	5.23	59020	34	74	10
40	646	P17661	Desmin	DESM_HUMAN	5.21	53560	80	251	41
41	1679	P17661	Desmin	DESM_HUMAN	5.21	53560	46	104	23

42	696	P02768	Albumin	ALBU_HUMAN	5.92	71317	51	120	31
43	1136	P51911	Calponin-1	CNN1_HUMAN	9.14	33321	50	78	20
44	761	P14618	Pyruvate kinase PKM	KPYM_HUMAN	7.96	58470	21	61	10
45	307	P63267	Actin, alpha cardiac muscle 1	ACTC_HUMAN	5.23	42334	74	169	29
46	785	P62736	Actin, aortic smooth muscle	ACTA_HUMAN	5.33	42381	39	130	21
47	723	P14618	Pyruvate kinase PKM	KPYM_HUMAN	7.96	58470	50	114	27
48	1636	Q01995	Transgelin	TAGL_HUMAN	8.87	22653	46	67	13
49	711	P25705	ATP synthase subunit alpha, mitochondria	ATPA_HUMAN	9.16	59828	41	82	22
50	209	O60304	Zinc finger protein 500	ZN500_HUMAN	6.84	54553	23	57	8
51	899	P04075	Fructose-bisphosphate aldolase A	ALDOA_HUMAN	8.30	39851	76	211	30
52	901	P63267	Actin, gamma-enteric smooth muscle	ACTH_HUMAN	5.31	42108	71	198	30
53	534	P17661	Desmin	DESM_HUMAN	5.21	53560	71	241	33
54	982	O00764	Pyridoxal kinase	PDXK_HUMAN	5.75	35308	57	129	23
55	1604	P62937	Peptidyl-prolyl cis-trans isomerase A	PPIA_HUMAN	7.68	18229	55	58	6
56	381	P02768	Albumin	ALBU_HUMAN	5.92	71317	12	57	7
57	843	P06733	Alpha-enolase	ENOA_HUMAN	7.01	47481	55	133	25
58	486	Q9UBX3	Mitochondrial dicarboxylate carrier	DIC_HUMAN	9.62	31718	32	58	8
59	1106	P02647	Apolipoprotein A-I	APOA1_HUMAN	5.56	30759	74	229	29
60	1283	P13645	Keratin, type I cytoskeletal 10	K1C10_HUMAN	5.13	59020	27	70	14
61	1083	Q5T5Y3	Calmodulin-regulated spectrin-associated protein 1	CAMP1_HUMAN	6.28	1792	20	63	6
62	208	P12110	Collagen alpha-2(VI)	CO6A2	5.85	109709	11	59	13

			chain						
63	998	P04075	Fructose-bisphosphate aldolase A	ALDOA	8.30	39851	31	69	9
64	695	P68032	Actin, alpha cardiac muscle 1	ACTC	5.31	42249	50	179	19
65	1270	P60174	Triosephosphate isomerase	TPIS	6.45	26938	79	195	21
66	1732	P07737	Profilin-1	PROF1	8.44	15216	34	77	8
67	1030	Q9NR45	Sialic acid synthase	SIAS	6.29	40738	43	98	17
68	369	P02545	Prelamin-A/C	LMNA	6.57	74380	32	179	19
69	775	P00352	Retinal dehydrogenase 1	AL1A1	6.30	55454	48	164	27
70	888	P08670	Vimentin	VIME	5.06	53676	69	201	34
71	870	P00558	Phosphoglycerate kinase 1	PGK1	8.30	44985	82	198	39
72	447	P02768	Albumin	ALBU	5.92	71317	42	129	22
73	1043	P14550	Aldo-keto reductase family 1 member A1	AK1A1	6.32	36892	35	66	9
74	1594	Q01995	Transgelin	TAGL	8.87	22653	70	101	19
75	977	P60709	Actin, cytoplasmic 1	ACTB	5.29	42052	57	207	27
76	607	P29401	Transketolase	TKT	7.58	68519	57	285	43
77	732	P25705	ATP synthase subunit alpha, mitochondrial	ATPA	9.16	59828	39	94	21
78	930	P06733	Alpha-enolase	ENOA	7.01	47481	21	61	7
79	1067	P17661	Desmin	DESM	5.21	53560	77	336	30
80	1618	Q08AG5	Zinc finger protein 844	ZN844	9.14	78708	31	72	15
81	313	P00966	Argininosuccinate synthase	ASSY	8.08	46786	19	59	9
82	896	P17661	Desmin	DESM	5.21	53560	45	124	12
83	231	P62937	Peptidyl-prolyl cis-trans isomerase A	PPIA	7.68	18229	89	118	18

84	905	P04406	Glyceraldehyde-3-phosphate dehydrogenase	G3P	8.57	36201	45	84	13
85	1599	P11532	Dystrophin	DMD	5.65	428979	11	61	8
86	1098	P21333	Filamin-A	FLNA	5.70	283301	19	86	11
87	1046	P25705	ATP synthase subunit alpha, mitochondrial	ATPA	9.16	59828	51	155	24

[a] Spot numbers.

[b] Protein accession number for SWISSPROT Database.

[c] Theoretical isoelectric point.

[d] Theoretical relative mass.

[e] MASCOT score

Table S4. Experimental conditions of the selected proteins' signature peptides used for proteomics validation.

Accession Number	Protein Name	Peptide Sequence [location]	Length (AA)	Molecular weight (g/mol)	MRM Transition		Cone voltage (v)	Collision energy (v)	RT (min)
					MS1 (m/z) (z)	MS2 (m/z) (ion type)			
P02545	Prelamin-A/C (LMNA)	K. AAYEAELGDAR.K [79,89]	11	1165.5	583.3 (+2)	860.4 (y8)<br>731.3 (y7)	35	21	3.35
P51911	Calponin-1 (CNN1)	R. EWIEGVTGR.R [36,44]	9	1045.5	523.8 (+2)	618.3 (y6)<br>731.4 (y7)	35	18	4.12
P68104	Elongation factor 1-alpha 1 (EF1A1)	K.AVFPLLYVR.R [632,640]	9	1077.6	539.3 (+2)	663.4 (y5)<br>760.5 (y6)	35	19	4.89
P04075	Fructose-bisphosphate aldolase A(ALDOA)	R.QLLLTADDR.V [61,69]	9	1044.6	522.8 (+2)	803.4 (y7)<br>690.3 (y6)	35	18	5.55
P25705	ATP synthase subunit alpha, mitochondrial (ATPA)	R.TGAIVDVPVGEELLGR.V [134,149]	16	1624.9	812.9 (+2)	1068.6 (y10)<br>969.5 (y9	35	29	5.46
P07355	Annexin A2 (ANXA2)	K.GVDEVTIVNILTNR.S [50,63]	14	1542.9	771.9 (+2)	1043.6 (y9)<br>942.6 (y8)	35	27	6.21
